# Systematic Methodological Evaluation of a Multiplex Bead-Based Flow Cytometry Assay for Detection of Extracellular Vesicle Surface Signatures

**DOI:** 10.3389/fimmu.2018.01326

**Published:** 2018-06-13

**Authors:** Oscar P. B. Wiklander, R. Beklem Bostancioglu, Joshua A. Welsh, Antje M. Zickler, Florian Murke, Giulia Corso, Ulrika Felldin, Daniel W. Hagey, Björn Evertsson, Xiu-Ming Liang, Manuela O. Gustafsson, Dara K. Mohammad, Constanze Wiek, Helmut Hanenberg, Michel Bremer, Dhanu Gupta, Mikael Björnstedt, Bernd Giebel, Joel Z. Nordin, Jennifer C. Jones, Samir EL Andaloussi, André Görgens

**Affiliations:** ^1^Clinical Research Center, Department of Laboratory Medicine, Karolinska Institutet, Stockholm, Sweden; ^2^Evox Therapeutics Limited, Oxford, United Kingdom; ^3^Molecular Immunogenetics and Vaccine Research Section, Vaccine Branch, Center for Cancer Research, National Cancer Institute, National Institutes of Health, Bethesda, MD, United States; ^4^Division of Pathology F56, Department of Laboratory Medicine, Karolinska Institutet, Karolinska University Hospital, Stockholm, Sweden; ^5^Institute for Transfusion Medicine, University Hospital Essen, University of Duisburg-Essen, Essen, Germany; ^6^Department of Clinical Neuroscience, Karolinska Institutet, Karolinska University Hospital, Stockholm, Sweden; ^7^Department of Biology, College of Science, Salahaddin University-Erbil, Erbil, Iraq; ^8^Department of Otorhinolaryngology & Head/Neck Surgery, University Hospital Düsseldorf, Heinrich Heine University of Düsseldorf, Düsseldorf, Germany; ^9^Department of Pediatrics III, University Children’s Hospital of Essen, University Duisburg-Essen, Essen, Germany; ^10^Department of Physiology, Anatomy and Genetics, University of Oxford, Oxford, United Kingdom

**Keywords:** exosomes, microvesicles, extracellular vesicles, extracellular vesicle flow cytometry, bead-based flow cytometry, exosome analysis, liquid biopsy, extracellular vesicle surface signature

## Abstract

Extracellular vesicles (EVs) can be harvested from cell culture supernatants and from all body fluids. EVs can be conceptually classified based on their size and biogenesis as exosomes and microvesicles. Nowadays, it is however commonly accepted in the field that there is a much higher degree of heterogeneity within these two subgroups than previously thought. For instance, the surface marker profile of EVs is likely dependent on the cell source, the cell’s activation status, and multiple other parameters. Within recent years, several new methods and assays to study EV heterogeneity in terms of surface markers have been described; most of them are being based on flow cytometry. Unfortunately, such methods generally require dedicated instrumentation, are time-consuming and demand extensive operator expertise for sample preparation, acquisition, and data analysis. In this study, we have systematically evaluated and explored the use of a multiplex bead-based flow cytometric assay which is compatible with most standard flow cytometers and facilitates a robust semi-quantitative detection of 37 different potential EV surface markers in one sample simultaneously. First, assay variability, sample stability over time, and dynamic range were assessed together with the limitations of this assay in terms of EV input quantity required for detection of differently abundant surface markers. Next, the potential effects of EV origin, sample preparation, and quality of the EV sample on the assay were evaluated. The findings indicate that this multiplex bead-based assay is generally suitable to detect, quantify, and compare EV surface signatures in various sample types, including unprocessed cell culture supernatants, cell culture-derived EVs isolated by different methods, and biological fluids. Furthermore, the use and limitations of this assay to assess heterogeneities in EV surface signatures was explored by combining different sets of detection antibodies in EV samples derived from different cell lines and subsets of rare cells. Taken together, this validated multiplex bead-based flow cytometric assay allows robust, sensitive, and reproducible detection of EV surface marker expression in various sample types in a semi-quantitative way and will be highly valuable for many researchers in the EV field in different experimental contexts.

## Introduction

Extracellular vesicles (EVs) can be harvested from cell culture supernatants and from all body fluids. They can be roughly classified based on their size and subcellular origin as exosomes (70–150 nm in diameter) which are released when multivesicular bodies fuse with the plasma membrane (1), or microvesicles (100 nm to 1 µm in diameter) which are formed by the outward budding of the plasma membrane (2, 3). In addition to these different EV subtypes, nowadays it is accepted in the field that there is likely to be a much higher degree of EV heterogeneity at multiple levels also within each subentity [reviewed in Ref. (4)]. As of now, no specific surface markers discriminating exosomes from microvesicles have been identified, and only a few EV surface markers have been reliably linked to specific cell sources. However, there is accumulating evidence that the protein composition and surface signature of EVs is likely dependent on the cell-type releasing them, the cell’s activation status and multiple other parameters. It can also be assumed that single cells release several functionally and phenotypically different types or classes of EVs (5–12). In general, addressing questions of heterogeneity in EV-containing samples has been challenging, mainly due to the small size of EVs and the lack of qualified, robust, and rapid methods to analyze multiple parameters of single EVs. However, the identification of specific vesicular surface markers will be of great relevance to further understand the molecular content and related functions of subsets of EVs, to identifying potential EV subsets with a defined therapeutic activity, and to uncovering and defining specific disease-related biomarkers.

Particularly within recent years, technical advancements have led to the development of new approaches enabling the analysis of EVs at the single vesicle level [reviewed in Ref. (13)]. Many of those methods are based upon light scattering, electron microscopy, fluorescence detection, or structural analysis (12, 14–17). Furthermore, a plethora of dedicated flow cytometric approaches have been developed and refined for single EV counting and phenotyping (6, 18–24), or for single EV sorting (25, 26). Several guidelines and protocols have been published in recent years with the aim to educate researchers and make the field aware of potential pitfalls, measurement artifacts like swarm detection and background caused by antibodies or lipoproteins (20, 21, 27–40). However, the widespread application and use of single EV analysis by flow cytometric methods is still hampered by the above-mentioned challenges, pitfalls, and ambiguities, and by the limited availability of appropriate instrumentation. Furthermore, single EV analysis requires time-consuming operations which in turn require extensive flow cytometric expertise for sample preparation, acquisition, and data analysis.

To overcome such issues, relatively simple bead-based protocols relying on the capture of EVs on antibody-coated beads with flow cytometric read-outs have been used to probe for the presence of candidate EV surface markers (22, 41–45). Of note, a recent validation study of a bead-based protocol showed a clear correlation between mean fluorescence intensities and EV contents (46). Moreover, a bead-based assay including 39 different antibody-coated multiplexed bead populations was recently described and used to assess and identify EV surface markers with clear differential expression between different blood cell type EVs by using conventional flow cytometry (10).

Here, we have critically investigated this novel multiplex bead-based flow cytometric assay and hereby present its methodological optimization and validation. During the course of our experiments we have optimized and explored different sample- and assay-related parameters in terms of detection limit, range of detection and reproducibility. We further provide different kinds of experimental examples to address basic but essential parameters for the assessment of EV surface signatures such as differential EV isolation protocols and storage conditions. Thus, we show that this multiplex bead-based flow cytometric assay, which could be applied in most laboratories, allows for reproducible detection of EV surface marker expression in various EV-containing sample types in a semi-quantitative manner. We conclude that this now validated assay will help EV researchers and support new discoveries in different areas of interest in the EV field.

## Materials and Methods

### Cells and Cell Culture

Unless indicated otherwise, cell lines were cultured in the following media: HEK293T cells were cultured in DMEM (containing Glutamax-I and sodium pyruvate; 4.5 g/L glucose; Invitrogen) supplemented with 10% FBS (Invitrogen), 1× Antibiotic-Antimycotic (Anti-Anti; ThermoFisher Scientific). Immortalized, human bone marrow-derived mesenchymal stromal cells [hTert + mesenchymal stromal cell line (MSCs)] (47) were cultured in RPMI-1640 (containing Glutamax-I and 25 mM HEPES; Invitrogen) supplemented with 10% FBS (Invitrogen), 10^−6^ mol/L hydrocortisone (Sigma) and 1× Anti-Anti. PANC-1 cells (48) were cultured in DMEM/F12 (containing 2.5 mM l-glutamine, 15 mM HEPES) supplemented with 10% heat-inactivated FBS (Invitrogen). IGROV1 cells (49) were cultured in RPMI-1640 (containing Glutamax-I and 25 mM HEPES; Invitrogen) supplemented with 10% heat-inactivated FBS (Invitrogen). All cell lines were grown at 37°C, 5% CO_2_ in a humidified atmosphere. For some experiments, media was changed to OptiMEM (Invitrogen) 48 h before harvest of conditioned media (CM) as described before (50). Unless indicated otherwise, all CM samples were directly subjected to a low speed centrifugation step at 500 × *g* for 5 min followed by a 2,000 × *g* spin for 10 min to remove larger particles and cell debris. FOLR1 cell surface expression on PANC-1 and IGROV1 cell lines was assessed by staining with APC-conjugated anti-human FOLR1 monoclonal antibodies (R&D Systems, clone 548908) *via* flow cytometry. Further details on sample processing are provided in Table S2 in Supplementary Material.

### Isolation and Culture of Human Hematopoietic Progenitor Cell Subsets

Human umbilical cord blood (UCB) was obtained from donors at the University Hospital Essen, Germany, after informed written consent according to the Declaration of Helsinki. The experimental usage of UCB samples was approved by the local ethics commission. Mononuclear cells were isolated by Ficoll (Biocoll Separating Solution, Biochrom) density gradient centrifugation and highly enriched for human hematopoietic CD34+ stem/progenitor cells as described previously (51, 52). For flow cytometric cell sorting of MPP-, LMPP-, and EMP-enriched hematopoietic progenitor subfractions, freshly isolated CD34+ cells were labeled with the following antibodies: anti-CD34-APC-AF750 (Beckman Coulter, clone 581), anti-CD45-BV510 (BD Biosciences, clone HI30), anti-CD133/1-APC (Miltenyi Biotec, clone AC133), anti-CD45RA-BV711 (BioLegend, clone HI100), and anti-CD38-BV786 (BD Biosciences, clone HIT2) antibodies as described before (52). Dead cells were excluded by 7-AAD (Beckman Coulter) staining. Cells were sorted using a FACSAria IIIu cell sorter (BD Biosciences) to a purity above 99.5%. Sorted cells were seeded at a density of 25,000 cells/300 μL in 48-well plate and cultured in a humidified atmosphere at 37°C and 5% CO_2_ in IMDM (Lonza) supplemented with 20% FBS (Biochrom), 100 U/mL penicillin, and 100 U/mL streptomycin (Life Technologies) and with FLT3L, SCF, and TPO each at 10 ng/mL final concentration (all Miltenyi Biotec). CM were harvested after 4 days. Further information on sample processing and storage is provided in Table S2 in Supplementary Material.

### Cerebral Spinal Fluid (CSF) Samples

Cerebral spinal fluid samples included in this study were derived from patients who underwent a lumbar puncture for clinical purposes at Neurology department at Karolinska University Hospital, Stockholm Huddinge, Sweden. Written informed consent was obtained from all subjects in accordance with the Declaration of Helsinki. This study was approved by the Regional Ethical Review Board in Stockholm, Sweden. All CSF samples were pre-cleared by 400 × *g* for 10 min and subsequent 2,000 × *g* centrifugation for 10 min, and filtered through 0.22 µm syringe filters with cellulose acetate membrane (VWR). Further information on sample processing and storage is provided in Table S2 in Supplementary Material.

### Human Blood Samples

The prospective clinical studies 02-C-0064, 04-C-0257, and 09-C-0195 were approved by the Institutional Review Board of the National Cancer Institute (NCI; MD, USA). Informed consent was obtained from all donors. For the data presented in this study, plasma and serum samples were processed as follows: 6 mL samples of blood from healthy volunteers were isolated in heparin and serum-separating tubes. The blood was spun at 2,500 × *g* for 20 min twice with the platelet poor plasma being isolated. Samples were then either frozen at –80°C or kept at 4°C, and run through size exclusion chromatography (SEC) columns (single qEV columns, IZON, New Zealand) according to the manufacturer’s recommendations were indicated.

### Mouse Experiments

Female NMRI mice with a bodyweight around 20 g were intravenously (tail vein) injected with 2 × 10^11^ hTert + MSC-EVs in 100 µL PBS. Blood was sampled by heart puncture 1 min and 30 min after injection and collected into PST-tubes (BD Biosciences) according to manufacturer’s instructions. Samples were depleted from cells by centrifugation at 2,000 × *g* for 10 min. Plasma samples were subjected to 0.22 µm filtration, and 120 µL filtered plasma was transferred to the MACSPlex Exosome assay. The animal experiments were approved by the Swedish Local Board for Laboratory Animals. The experiments were performed in accordance with the ethical permissions granted, and designed to minimize the suffering and pain of the animals.

### Isolation of EVs From Cell Culture Supernatant

Several different EV isolation protocols, and variations or combinations thereof, were applied in this study in order to compare the detectable EV surface signatures in respective fractions with the MACSPlex Exosome flow cytometry assay. See Table S2 in Supplementary Material for detailed information how EVs were prepared for which experiment. Generally, CM was pre-cleared first by a low speed centrifugation step (500–900 × *g* for 10 min) followed by centrifugation at 2,000 × *g* for 10–20 min to remove larger particles and debris. Unless indicated otherwise, samples were subsequently filtered through syringe (VWR) or bottle top filters (Corning, low protein binding) with cellulose acetate membranes (0.22 µm pore size) to remove any larger particles. To purify EVs with differential UC CM samples were either first subjected to centrifugation at 10,000 × *g* for 30 min or directly subjected to UC at 110,000 × *g* for 90 min to pellet the EVs. A second washing step was performed in both cases by resuspending the EV pellet in 25 mL of PBS and another 90 min of UC at 110,000 × *g*. 10,000 and 110,000 × *g* centrifugation steps were performed at 4°C using the Beckman Coulter Type 70 Ti rotor in a Beckman Coulter L-80 ultracentrifuge. To concentrate EVs *via* tangential flow filtration (TFF) CM was diafiltrated with at least two times of the initial volume of PBS and concentrated to 20 mL using the KR2i TFF system (SpectrumLabs) equipped with modified polyethersulfone hollow fiber filters with 300 kDa membrane pore size (MidiKros, 370 cm^2^ surface area, SpectrumLabs) at a flow rate of 100 mL/min (transmembrane pressure at 3.0 psi and shear rate at 3,700 s^−1^). To concentrate CSF samples with starting volumes of 20–30 mL, smaller versions of the same filter type (MicroKross, 20 cm^2^, SpectrumLabs) were used to diafiltrate and concentrate samples down to 1 mL manually. To further purify EVs *via* bind-elute SEC (BE-SEC) pre-concentrated CM samples were loaded onto BE-SEC columns (HiScreen Capto Core 700 column, GE Healthcare Life Sciences), connected to an ÄKTAstart chromatography system (GE Healthcare Life Sciences) as described previously (50). All settings were chosen according to the manufacturer’s instructions. Briefly, the EV sample was collected according to the 280 nm UV absorbance chromatogram and concentrated to a final volume of 500 µL by using an Amicon Ultra-15 10 kDa molecular weight cut-off spin-filter (Millipore).

### Nanoparticle Tracking Analysis (NTA)

Nanoparticle tracking analysis (15, 53) was applied to determine particle size and concentration of all samples. All plasma/serum samples were characterized by NTA with a NanoSight LM10 instrument (Malvern, UK), equipped with a 405 nm LM12 module and EMCCD camera (DL-658-OEM-630, Andor). Video acquisition was performed with NTA software v3.2, using a camera level of 14. Three 30 s videos were captured per sample. Post-acquisition video analysis used the following settings: minimum track length 5, detection threshold 4, automatic blur size 2-pass, maximum jump size 12.0. All other samples were characterized with a NanoSight NS500 instrument equipped with NTA 2.3 analytical software and an additional 488 nm laser. At least five 30 s videos were recorded per sample in light scatter mode with a camera level of 11–13. Software settings for analysis were kept constant for all measurements (screen gain 10, detection threshold 7). All samples were diluted in 0.22 µm filtered PBS to an appropriate concentration before analysis.

### Western Blotting

HEK293T cells and hTERT + MSCs were collected and counted using trypan blue 0.4% (Invitrogen, Thermo Fisher Scientific) in a Countess II FL automated cell counter (Invitrogen, Thermo Fisher Scientific). 2 × 10^6^ cells were pelleted at 300 × *g* for 5 min, washed once with cold PBS and pelleted at 300 × *g* for 5 min. The cell pellet was lysed with 100 µL of RIPA buffer, kept on ice, and vortexed five times every 5 min. The cell lysate was then spun at 12,000 × *g* for 10 min at 4°C and the supernatant was transferred to a new tube and kept on ice. Cells and particles were mixed with buffer containing 0.5 M dithiothreitol, 0.4 M sodium carbonate (Na_2_CO_3_), 8% SDS, and 10% glycerol, and heated at 65°C for 5 min. The samples were loaded onto a NuPAGE Novex 4–12% Bis-Tris Protein Gel (Invitrogen, Thermo Fisher Scientific) and run at 120 V in NuPAGE MES SDS running buffer (Invitrogen, Thermo Fisher Scientific) for 2 h. The proteins on the gel were transferred to an iBlot nitrocellulose membrane (Invitrogen, Thermo Fisher Scientific) for 7 min using the iBlot system. The membrane was blocked with Odyssey blocking buffer (LI-COR) for 60 min at RT with gentle shaking. After blocking, the membrane was incubated overnight at 4°C or 1 h at RT with primary antibody solution [1:1,000 dilution for anti-Alix (ab117600, Abcam) and anti-Tsg101 (ab30871, Abcam); 1:2,000 dilution for anti-CD9 (ab92726, Abcam)]. The membrane was washed with PBS supplemented with 0.1% Tween-20 (PBS-T, Sigma) five times for every 5 min and incubated with the corresponding secondary antibody (LI-COR) for 1 h at RT (1:15,000 goat anti-mouse IRDye800CW or 680LT to detect Alix; 1:15,000 dilution goat/anti-rabbit IRDye800CW or 680LT to detect CD9, Tsg101). The membrane was washed with PBS-T for five times within 25 min, twice with PBS and visualized on the Odyssey infrared imaging system (LI-COR).

### Generation of Stable Cell Lines

Codon-optimized DNA sequences coding for human CD63 (Uniprot accession number P08962) and the fluorescent proteins mNeonGreen (54) (GenBank accession number AGG56535.1), mCardinal (55) (GenBank accession number KJ131552), E2-Crimson (56) and Cerulean (57) (GenBank accession number AJD87366.1) were synthesized (Integrated DNA Technologies) as gene fragments and cloned downstream of the CAG promoter into the pLEX vector backbone using EcoRI and NotI. To generate the different constructs expressing respective fluorescent proteins fused to the C-terminus of CD63, fluorescent protein coding sequences (CDS) were subcloned into pLEX-CD63 using SacI and NotI. Next, the complete CDS of the different CD63-fluorescent protein fusions were cloned into the lentiviral p2CL9IPwo5 backbone downstream of the SFFV promoter using EcoRI and NotI, and upstream of an internal ribosomal entry site-puromycin resistance cDNA cassette (see Figure 5A). All expression cassettes were confirmed by sequencing. Lentiviral supernatants were produced as described previously (58). In brief, HEK293T cells were co-transfected with p2CL9IPw5 plasmids containing CD63 fused to the respective fluorescent proteins, the helper plasmid pCD/NL-BH, and the human codon-optimized foamyvirus envelope plasmid pcoPE (59–61) using the transfection reagent JetPEI (Polyplus, Illkrich Cedex). 16 h post transfection gene expression from the human CMV immediate-early gene enhancer/promoter was induced with 10 mM sodium butyrate (Sigma-Aldrich) for 6 h before fresh media was added to the cells, and the supernatant was collected 22 h later. Viral particles were pelleted at 25,000 × *g* for 90 min at 4°C. The supernatant was discarded and the pellet was resuspended in 2 mL of Iscove’s Modified Dulbecco’s Media supplemented with 20% FBS and 1% P/S. Aliquots were stored at −80°C until usage. To generate stable cell lines, HEK293T cells were transduced by overnight exposure to virus stocks and passaged at least five times under puromycin selection (Sigma; 6 µg/mL). The expression of respective CD63-fluorescent protein fusion constructs was confirmed *via* flow cytometry and fluorescence microscopy for all established cell lines (data not shown).

### Bead-Based Multiplex Exosome Flow Cytometry Assay

Different sample types were subjected to bead-based multiplex EV analysis by flow cytometry (MACSPlex Exosome Kit, human, Miltenyi Biotec) (9, 10), with details regarding sample preparation and normalization summarized in Table S2 in Supplementary Material. Unless indicated otherwise, EV-containing samples were processed as follows: Samples were diluted with MACSPlex buffer (MPB) to, or used undiluted at, a final volume of 120 µL and loaded onto wells of a pre-wet and drained MACSPlex 96-well 0.22 µm filter plate before 15 µL of MACSPlex Exosome Capture Beads (containing 39 different antibody-coated bead subsets) were added to each well. Generally, particle counts quantified by NTA, and not protein amount, were used to estimate input EV amounts. Filter plates were then incubated on an orbital shaker overnight (14–16 h) at 450 rpm at room temperature protected from light. To wash the beads, 200 µL of MPB was added to each well and the filter plate was put on a vacuum manifold with vacuum applied (Sigma-Aldrich, Supelco PlatePrep; −100 mBar) until all wells were drained. For counterstaining of EVs bound by capture beads with detection antibodies, 135 µL of MPB and 5 µL of each APC-conjugated anti-CD9, anti-CD63, and anti-CD81 detection antibody were added to each well and plates were incubated on an orbital shaker at 450 rpm protected from light for 1 h at room temperature. Next, plates were washed by adding 200 µL MPB to each well followed by draining on a vacuum manifold. This was followed by another washing step with 200 µL of MPB, incubation on an orbital shaker at 450 rpm protected from light for 15 min at room temperature and draining all wells again on a vacuum manifold. Subsequently, 150 µL of MPB was added to each well, beads were resuspended by pipetting and transferred to V-bottom 96-well microtiter plate (Thermo Scientific). Flow cytometric analysis was performed, unless indicated otherwise, with a MACSQuant Analyzer 10 flow cytometer (Miltenyi Biotec; see Table S1 in Supplementary Material for acquisition parameters) by using the built-in 96-well plate reader. All samples were automatically mixed immediately before 70–100 µL were loaded to and acquired by the instrument, resulting in approximately 7,000–12,000 single bead events being recorded per well. FlowJo software (v10, FlowJo LLC) was used to analyze flow cytometric data. Median fluorescence intensity (MFI) for all 39 capture bead subsets were background corrected by subtracting respective MFI values from matched non-EV buffer or media controls that were treated exactly like EV-containing samples (buffer/medium + capture beads + antibodies). GraphPadPrism 6 (GraphPadPrism Software, La Jolla, CA, USA) was used to analyze data and assemble figures. To generate heatmaps of data, flow cytometric data were gated in FlowJo with gated data being exported to comma separated files, which were subsequently imported into MATLAB (v9.3.0, Mathworks Inc.) for further analysis and data visualization. In order to compare data from the MACSQuant and FACS Symphony flow cytometers, the log10 transformed ratios of capture beads + EVs + Ab over their respective controls (capture beads + ab) was compared, rather than using background subtraction, which allowed for comparison despite axis scaling differences.

## Results and Discussion

### Detection of EV Surface Signatures With a Multiplex Bead-Based Flow-Cytometry Assay

In this study, we aimed to systematically evaluate and explore the capabilities of a recently described (10) multiplex bead-based flow cytometry assay platform for EV research. In its current form, this assay comprises 39 hard-dyed capture bead populations (4 µm diameter), each of them coated with different monoclonal antibodies against 37 potential EV surface antigens or two internal isotype negative controls. All bead populations can be identified and gated based on their respective fluorescence intensity according to the assay documentation provided by the manufacturer (Figure S1 in Supplementary Material). After incubation with EV-containing samples, bulk bead-captured EVs can subsequently be detected by counterstaining with APC-labeled detection antibodies against the tetraspanins CD9, CD63, and CD81, which are often referred to as common EV surface markers. In this study, we mostly used a mixture of all three antibodies (pan tetraspanin) in order to cover most EVs being present in respective samples. This assay hence relies on the detection of single capture beads, whereby each antibody-coated bead can capture multiple EVs. Bead-captured EVs for each bead, and subsequently for each bead population, can then indirectly be detected through the cumulative signal of multiple fluorescence-conjugated antibodies that bind to respective epitopes on the bulk bead-captured EVs.

**Figure 1:**
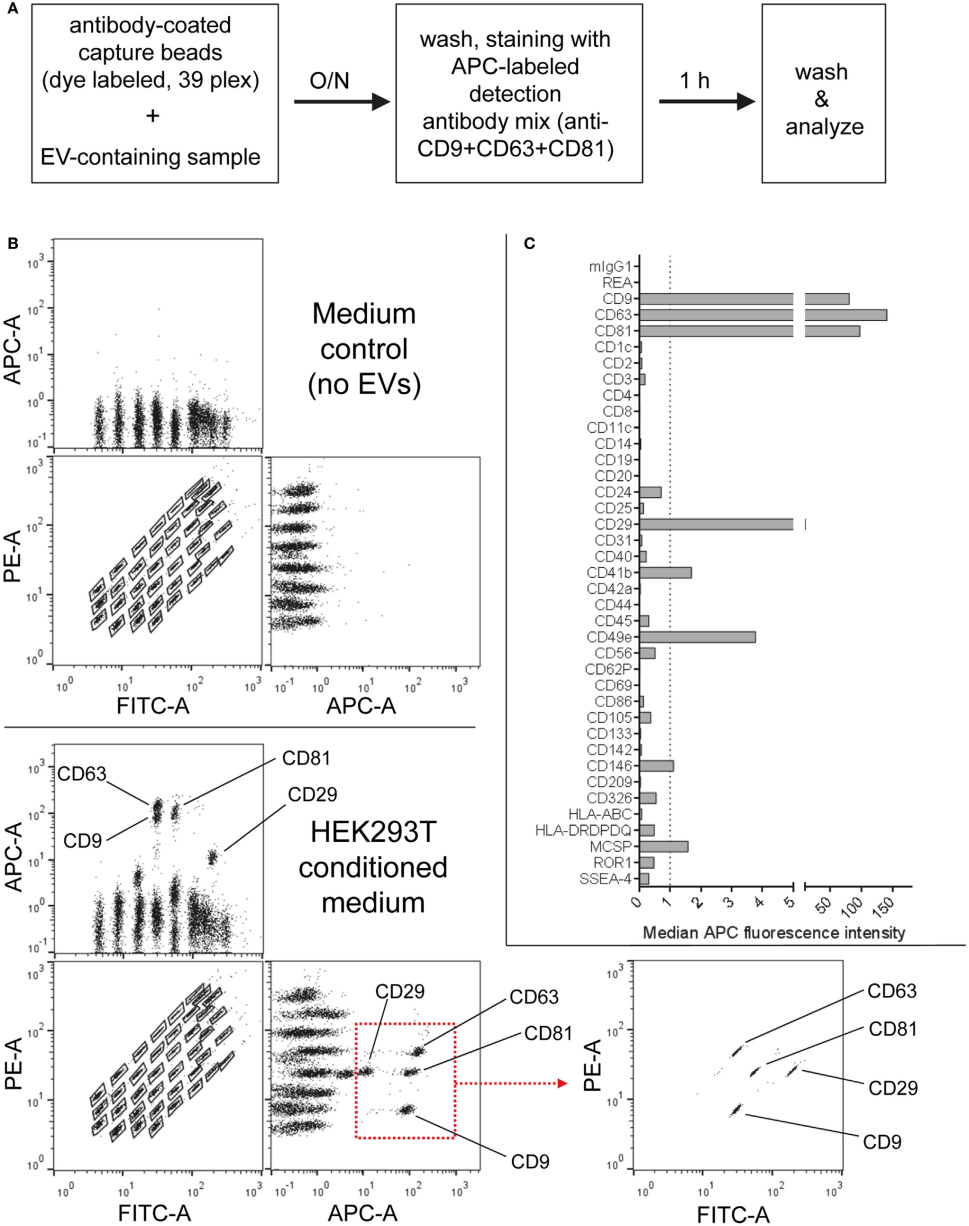
Multiplex bead-based flow cytometry assay principle for detection of extracellular vesicle (EV) surface signatures. **(A)** Overview of assay workflow. 39 multiplexed populations of dye-labeled antibody-coated capture beads are incubated with EV-containing samples. In this case, captured EVs are counterstained with APC-labeled detection antibodies by using a mixture of anti-CD9, anti-CD63, and anti-CD81 (pan tetraspanin) antibodies. **(B)** Results after analyzing HEK293T conditioned medium (CM) compared to the respective medium control, showing all 39 bead populations identified by their fluorescence in the FITC Vs. PE channel (see Table S1 in Supplementary Material) with adjunct dot plots showing respective APC-stained bead populations. Back-gating from the four bead populations with the brightest staining (CD9, CD29, CD63, and CD81) is shown as an example to underline the assay principle (bottom right). **(C)** Representative quantification of the median APC fluorescence values for all bead populations after background correction (medium control values subtracted from measured HEK293T CM values). See Figures S1 and S2 and Tables S1 and S2 in Supplementary Material for further details.

First, the EV content of HEK293T-derived pre-cleared CM was analyzed following overnight capture and pan tetraspanin detection (Figure 1A). Raw APC MFI values for each capture bead population were background corrected by subtracting corresponding MFI values obtained from media controls (capture beads + detection antibodies) subjected to the same protocol as samples (capture beads + EVs + detection antibodies; Figures 1B,C). The FITC and PE channels of a MACSQuant Analyzer 10 flow cytometer equipped with 405, 488, and 638 nm lasers were used to identify capture bead populations (Figure 1B; Figure S1 and Table S1 in Supplementary Material). When analyzing samples on flow cytometers equipped with additional green lasers, e.g., the Beckman Coulter Cytoflex S instrument, we observed a slightly different appearance of the bead populations when using respective channels designated for FITC Vs. PE detection (Figure S2A in Supplementary Material, left panel). However, by using more suitable filter sets for bead identification, a similar bead distribution to that from an instrument lacking a green laser could be achieved (Figure S2A in Supplementary Material, right panel).

Particularly four bead populations, i.e., CD9, CD63, CD81, and CD29, were detected as strongly positive in HEK293T CM, which was confirmed *via* backgating (Figures 1B,C). Other markers detected at intermediate- to low-positive APC fluorescence intensity levels comprised mainly CD24, CD41b, CD49e, CD146, and MCSP. Markers such as CD3, CD105, or CD326 were detected at very low levels after background correction (Figure 1C). Of note, most quantitative data in this study are plotted by using segmented linear axis scales, however, very low values might be better represented by logarithmic or alternatively segmented linear axis scales (Figure S2B in Supplementary Material). For improved comparability of plots with different axis scaling, we have included an arbitrary dotted line at an APC MFI value of 1 for all plots throughout this study, though it should be noted that this does not reflect an objective threshold for marker positivity.

These results confirm that this multiplex bead-based assay is sensitive enough to detect EV surface marker presence in pre-cleared, otherwise unfractionated CM samples. These data clearly indicate the expression of the abundant tetraspanin markers CD9, CD63, and CD81 and of the integrins CD29 and CD49e on HEK293T-derived EVs. Of note, this sandwich assay can only detect EVs that fulfill both of the following criteria: (1) EVs must be positive for at least one of the antigens detected by the antibody-coated capture bead populations, which include CD9, CD63, and CD81 and (2) EVs need to be positive for CD9, CD63, or CD81 when the pan tetraspanin detection cocktail is used. Generally, the results obtained from this assay could be influenced by several factors, including cross-linking of beads by single EVs binding to more than one bead population (see gating on single beads in Figure S1 in Supplementary Material), and thus should be interpreted not as a single vesicle quantification, but rather as a semi-quantitative bulk assessment of the general repertoire of EV surface marker expression, i.e., EV surface signatures, in an EV-containing sample. However, compared to Western blot, which similarly involves probing for antibody binding to one protein in a bulk format, this assay ultimately requires the binding of two antibodies to a single EV, which should result in more sensitive and more specific robust detection, while diminishing the possibility that a given positive signal is derived from free protein rather than intact EVs. Importantly, if the hypothetical surface markers A and B are detected as positive in this assay in a given sample, one cannot conclude if EVs in the sample are all positive for A and B, or if some are positive for A and negative for B and *vice versa*. Instead, this assay can be used to judge if a given marker is positive in a sample, while single EV analyses *via* dedicated flow cytometric assays would be required to detect EV heterogeneity within one sample. However, in contrast to most other more dedicated flow cytometry based methods for EV surface marker analysis, this multiplex bead-based assay can be run on most classical flow cytometers equipped with blue and red lasers and requires less-extensive expertise in flow cytometry.

### Evaluation of the Assays Range of Detection Through Assay Input Titration

**Figure 2:**
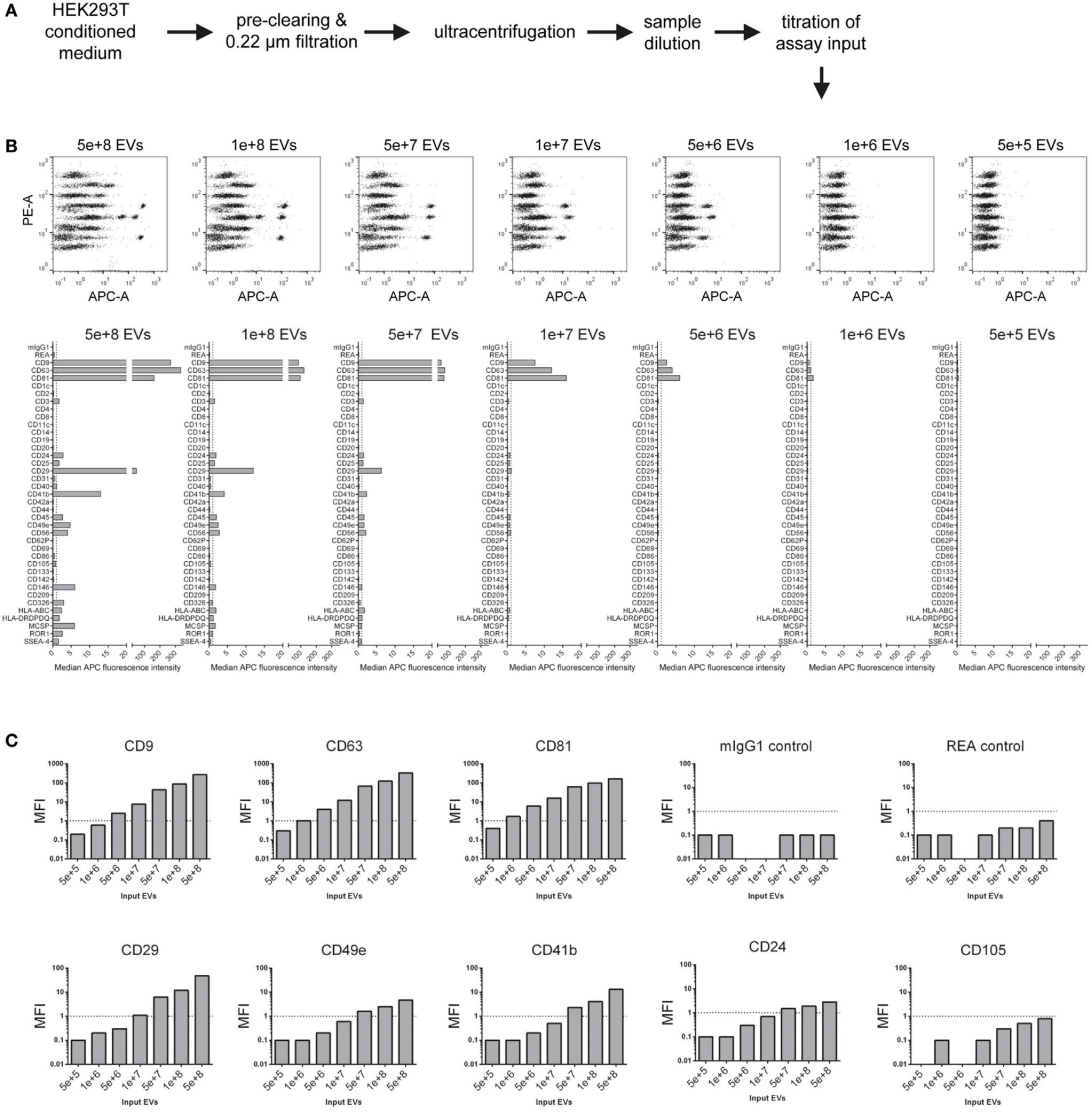
Evaluation of assay range of detection and titration of assay input. **(A)** Experimental outline. The concentration of HEK293T-derived extracellular vesicles (EVs) was quantified *via* nanoparticle tracking analysis, and defined doses of EVs were used as assay input. **(B)** Assay results shown for a range of 5 × 10^5^–5 × 10^8^ particles per assay. **(C)** Same experimental dataset as shown in **(B)** sorted by different markers Vs. isotype controls, demonstrating that more abundant markers (e.g., CD63) can be still detected at relatively low input doses, while less abundant markers (e.g., CD49e) require higher input doses, in this case above 1 × 10^8^ particles for reliable detection of signal above background levels. Further details are provided in Table S2 in Supplementary Material.

Considering the principle of this multiplex bead-based assay, the strength of any signal detected with APC-conjugated detection antibodies strongly depends on the number of EVs added to the assay. While previous reports relied on defining EV inputs by protein amounts (9, 10), the ratio of EVs to total protein content of a given sample will be dependent on the purity of the sample, which in turn can vary drastically depending on which isolation method was used and subsequently purity was achieved (63). Thus, we next aimed to define EV input numbers as particle counts based on NTA. EVs were isolated from HEK293T-derived CM with a differential centrifugation protocol which is classically used to enrich for exosomes (64) (Figure 2A; Table S2 in Supplementary Material). NTA-based calculated EV doses between 5 × 10^5^ and 5 × 10^8^ were used as input for the multiplex bead-based flow cytometry assay. As expected, signal intensities for all positively stained bead populations, but not internal isotype control bead populations, were decreasing with decreasing EV input, with almost no detectable signals at an input dose of 5 × 10^5^ EVs (Figures 2B,C). Though the presence of the tetraspanin markers CD9, CD63, and CD81 could be clearly detected already at doses of 5 × 10^6^ EVs, less abundant markers like CD29, CD41b, and CD49e required approximately 10-fold higher EV inputs for reliable detection. At the highest dose tested (5 × 10^8^), several markers are negative or near background at lower doses were detected as positive, e.g., CD24, CD146, MCSP, and ROR1, in addition to rather unexpected markers, e.g., the hematopoietic surface marker CD45, and the NK cell marker CD56 (NCAM) (Figures 2B,C).

This dataset shows that NTA-based EV quantification is suitable for defining EV input doses for this assay. These results further imply that the range of detection of this assay depends on the abundance of markers and the detail of information a given experiment aims to achieve. If semi-quantitative assessment of only highly abundant markers is needed for a given EV-containing sample, then the EV input can be less than for experiments aiming to cover the whole EV surface signature present in a sample. Furthermore, the minimum amount of EVs required will not only be dependent on the EV input dose but also on the abundance of the marker and the sensitivity of the flow cytometer used. Generally, it appears that an EV input between 1 × 10^8^ and 1 × 10^9^ should be suitable for cell culture media-derived EVs. However, given that different cells might release different quantities of EVs with different surface marker composition, unknown samples should be titrated or measured at different dilutions, if possible. In addition, this data further demonstrates that the lack of reliable detection of signal from a bead population does not necessarily mean that a certain marker is not expressed, it could just be expressed at such low levels, or only on a minor subset of EVs in a sample, such that the signal would be below the limit of detection of this assay, on the instrument used, for this sample. Conversely, signals for markers being detected at low levels close to background also may relate to unspecific binding or background, e.g., the above-mentioned detection of the hematopoietic marker CD45 on HEK293T EVs. On the other hand, if an EV surface marker is detected in this assay, and if its signal increases with increasing sample input, this strongly suggests that this marker is expressed on EVs in this sample.

### Evaluation of Assay Variability

**Figure 3:**
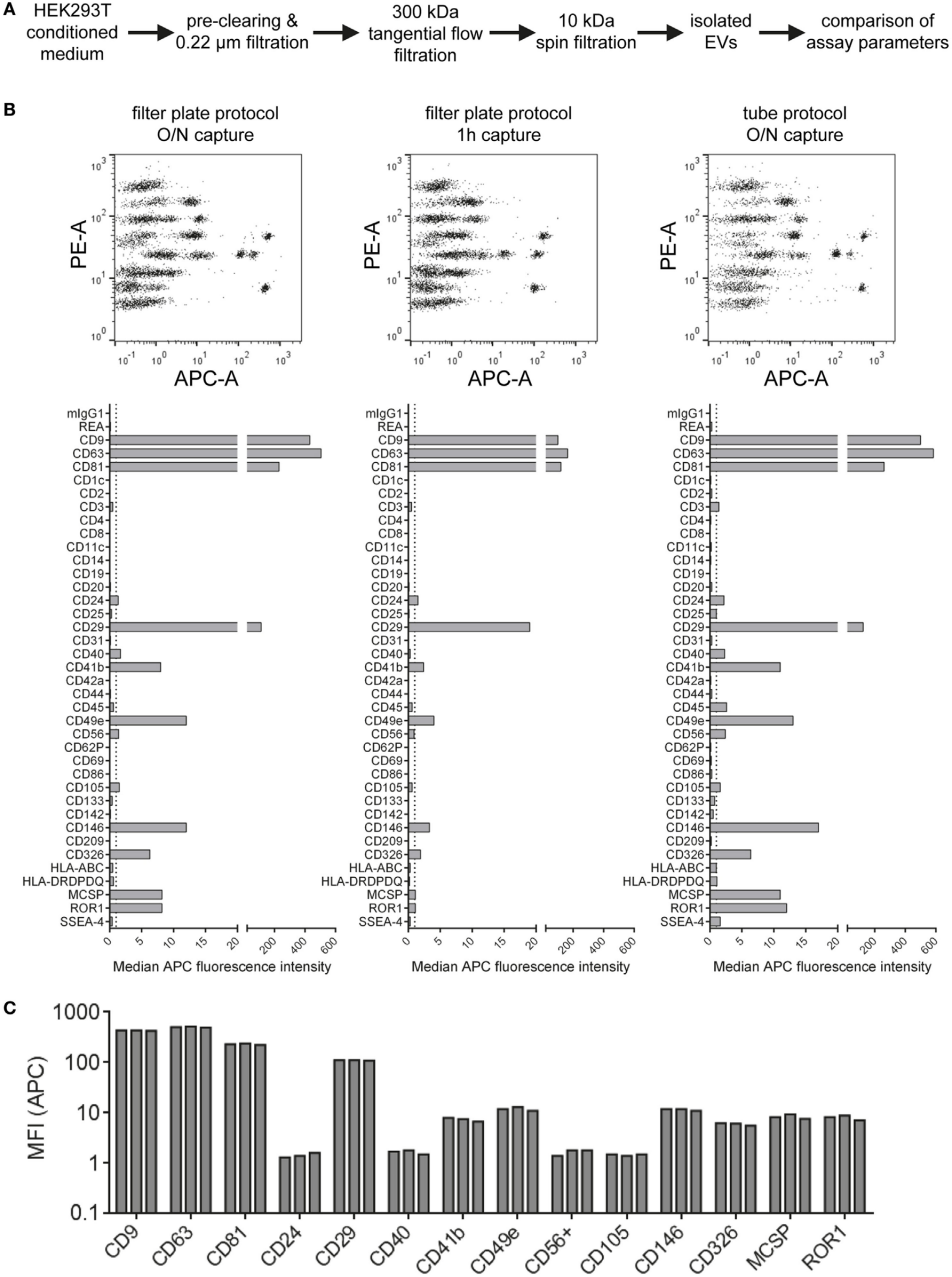
Evaluation of protocol parameters and assay variability. **(A)** Experimental outline. Isolated HEK293T extracellular vesicles were subjected to different assay protocols with input doses of 5 × 10^8^ particles/assay. **(B)** Assay results with the default protocol used throughout this study (filter plate protocol, O/N capture; left), a protocol with the capture time shortened to 1 h (middle) and a protocol performed in microcentrifuge tubes instead of filter plates but with O/N capture (right). **(C)** Marker intensities from three replicate measurements for most abundant markers (filter plate protocol, O/N capture). See Figure S3 in Supplementary Material for complete datasets of all replicates and Table S2 in Supplementary Material for further details.

Aiming to further standardize this assay and optimize assay parameters related to sample preparation and assay protocol, we next compared different basic assay protocols. HEK293T-derived EVs were isolated *via* ultrafiltration, more specifically TFF with subsequent 10 kDa spin filtration (Figure 3A; Table S2 in Supplementary Material). Subsequently, isolated EVs were analyzed at input doses of 5 × 10^8^ EVs per assay with three different protocols. The default protocol used throughout this study is based on 0.22 µm 96 well filter plates which are used for all steps including over-night incubation with capture beads, staining (1 h) with detection antibodies and washing steps. This default protocol was compared to a shortened protocol version (1 h incubation with capture beads instead of over-night) and to a protocol in which all steps were performed in standard microcentrifuge tubes instead of a filter plate. All volumes and EV/reagent amounts were otherwise kept constant, and measurements were done in triplicates for all three protocols in order to evaluate respective assay variability (Figure 3B; Figure S3 in Supplementary Material). When using the protocol with a shorter bead capture + EV incubation time, we generally observed lower APC MFI values for all positive bead populations when compared to over-night incubation. In contrast, performing all steps of the assay protocol in tubes rather than filter plates led to consistently higher APC MFI values for all detected markers (Figure 3B; Figure S3 in Supplementary Material). When comparing replicates done with the same respective protocol variant, highly consistent results with low variability were observed in all cases (Figure 3C; Figure S3 in Supplementary Material).

Generally, the results indicate that EV surface signature detection and quantification are relatively consistent and reproducible when assay parameters are kept constant, at least for EV samples derived from HEK293T cell culture supernatant. The trend toward lower MFI intensities detected when the capture bead incubation step is reduced from over-night to 1 h indicates incomplete binding of EVs to capture beads. However, abundant markers were still detectable and most EV surface marker signals were still comparable. Thus, this shortened protocol would be applicable for screening or experiments that do not require optimized detection. Though, if markers with lower abundance are to be detected accurately, the over-night incubation step would be preferred. The consistently increased signals detected when performing the assay protocol in tubes are probably related to different mixing conditions during the incubation steps or differences of bead adhesion to plastic surfaces. We further studied sample stability after completing the capture and staining protocol and obtained highly similar results when performing flow cytometric data acquisition directly or after storing the sample one week at 4°C, even though the total beads acquired for such measurements of leftover samples were lower in most cases (Figure S4 in Supplementary Material). This indicates that the time between assay preparation and data acquisition is not highly critical as long as the samples are kept at 4°C and protected from light. Taken together, all protocols applied are valid and lead to similar results, with robust and reproducible EV surface signature quantification independent of the protocol used for this multiplex bead-based assay.

### Stepwise Evaluation and Monitoring of Different EV Isolation Protocols

Our results indicate that this multiplex bead-based flow cytometry assay can be used to assess EV surface marker signatures in both unprocessed CM samples and in samples following EV enrichment by different isolation protocols (Figures 1–3; Table S2 in Supplementary Material). While the presence of abundant markers on HEK293T-derived EVs appeared rather consistent, we observed slight differences regarding their ratio when comparing CM (Figure 1), EVs isolated *via* differential ultracentrifugation (UC; Figure 2) and EVs isolated *via* ultrafiltration (Figure 3). For example, CD49e was detected at higher levels than CD41b in CM (Figure 1C) and filtration-isolated EVs (Figure 3B), while the opposite was observed in UC-isolated EVs (Figure 2B). Differential UC-based protocols are classically used in the field, and recently were reported to have potentially negative effects on the intactness of EVs (65–68). Thus, different alternative isolation protocols, e.g., based on TFF or SEC, have been established in recent years (50, 67, 69). Furthermore, there is accumulating evidence for the existence of different types of EVs in terms of density, size, and surface phenotype (10–12, 70). Since every EV isolation protocol might have a certain bias toward subsets of EVs, we next aimed to evaluate the use of this multiplex bead-based EV assay to analyze different EV-containing fractions throughout an isolation process.

**Figure 4:**
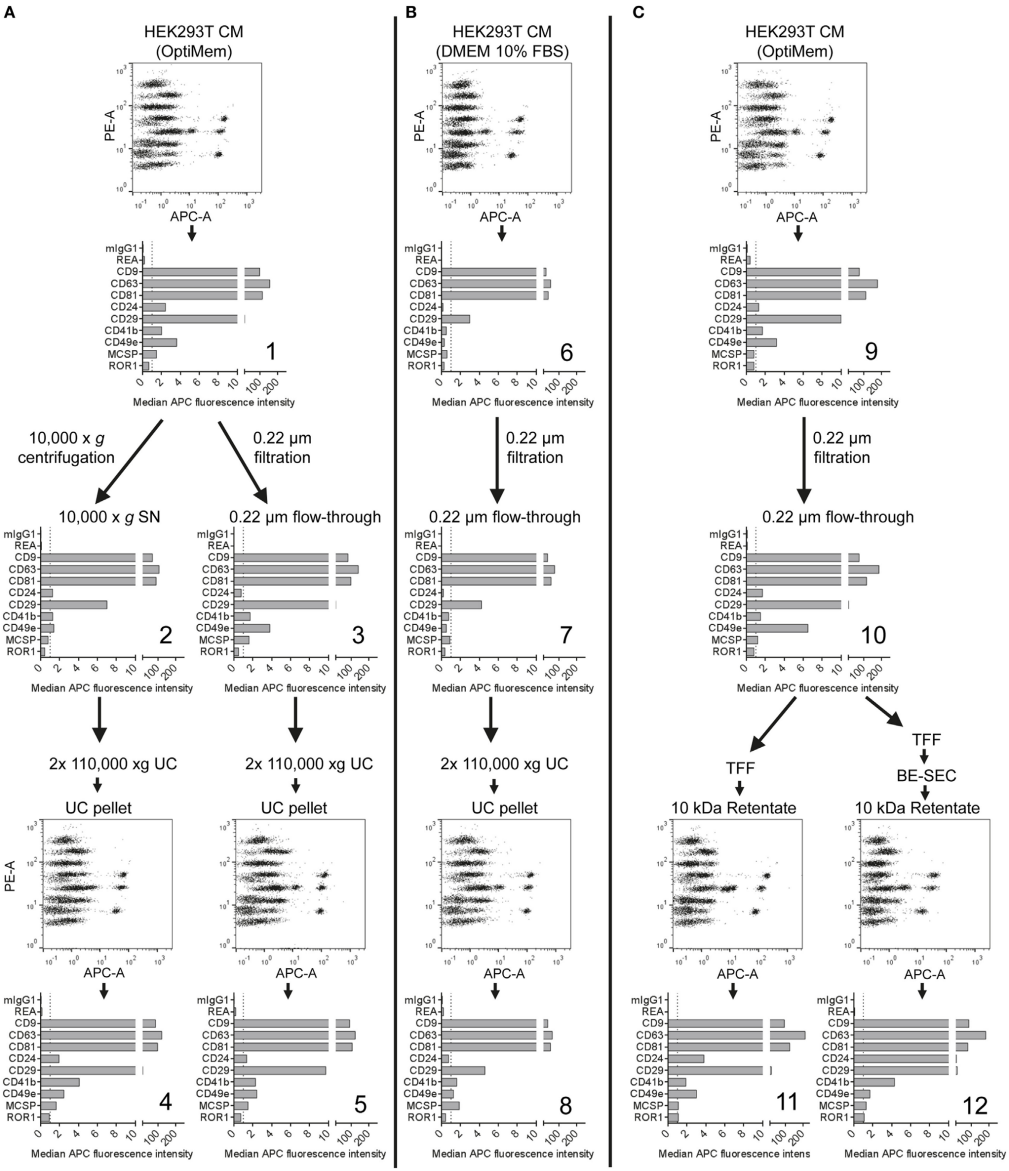
Assessment of extracellular vesicles (EV) surface signatures throughout different EV isolation protocols. Bead-based multiplex flow cytometry assay results for selected markers. Comparison of EV surface signatures of HEK293T-derived conditioned medium (CM) samples with samples during and after processing with different EV isolation protocols. **(A)** HEK293T cells were cultured under serum-free (SF) conditions and EVs were isolated *via* two different protocols based on differential ultracentrifugation (UC). **(B)** EVs were isolated with a differential UC-based protocol, starting from CM derived from HEK293T cells cultured in FBS-containing media. **(C)** HEK293T cells were cultured under SF conditions and EVs were isolated *via* tangential flow filtration, with and without inclusion of a bind-elute size exclusion chromatography (BE-SEC) clean-up step. See Figure S5 in Supplementary Material for complete datasets and Table S2 in Supplementary Material for further details. This figure shows one representative example out of two independently performed experiments.

HEK293T-derived CM was derived from cells incubated with either serum-free (SF; Figures 4A,C) or serum-supplemented (SS) medium (Figure 4B). Respective medium controls did not show substantial background signals, indicating that FBS-derived bovine EVs do not cross-react with the antibodies used in this assay (data not shown). Both CM samples were subjected to the same classical differential centrifugation protocol (Figures 4A,B), and SF CM was further processed by using a protocol based on TFF and subsequent bind-elute SEC (BE-SEC) which was recently described by our group (50). Samples were taken at different steps before, during and after the isolation process, and all samples were analyzed with the multiplex bead-based assay with standardized assay input doses of 1 × 10^8^ particles. Generally no major differences between samples were detected in terms of presence/absence of EV surface markers (Figure 4). Compared to SF cultures, SS CM samples and downstream fractions showed consistently lower signals for all markers (Figure 4B). This probably relates to the presence of FBS-derived EVs that affect NTA-based EV quantification, and/or that HEK293T cells secreted less EVs when cultured in this medium. Furthermore, it appeared that the 10,000 × *g* centrifugation step before UC resulted in a reduced abundance of CD49e per EV when compared to CM or 0.22 µm filtrated samples (Figures 4A,C). Results from samples purified *via* BE-SEC indicated enriched CD24 expression on EVs purified in this way, when compared to unpurified CM or samples post TFF only (Figure 4C). However, such minor differences in less abundant EV markers cannot be a valid basis to draw final conclusions on without further validation. Thus, based on this dataset we cannot conclude if, and which, isolation methods introduce distinctive bias toward certain phenotypic EV subsets. In summary, we can, however, conclude that the multiplex bead-based assay is suitable to assess the EV surface marker composition in various EV-containing samples with highly variable purities throughout an EV isolation process, indicating that this assay can also be valuable for in-process monitoring of EV isolation and fractionation protocols, or for screening.

### Assay Compatibility With Fluorescently Labeled EVs

**Figure 5:**
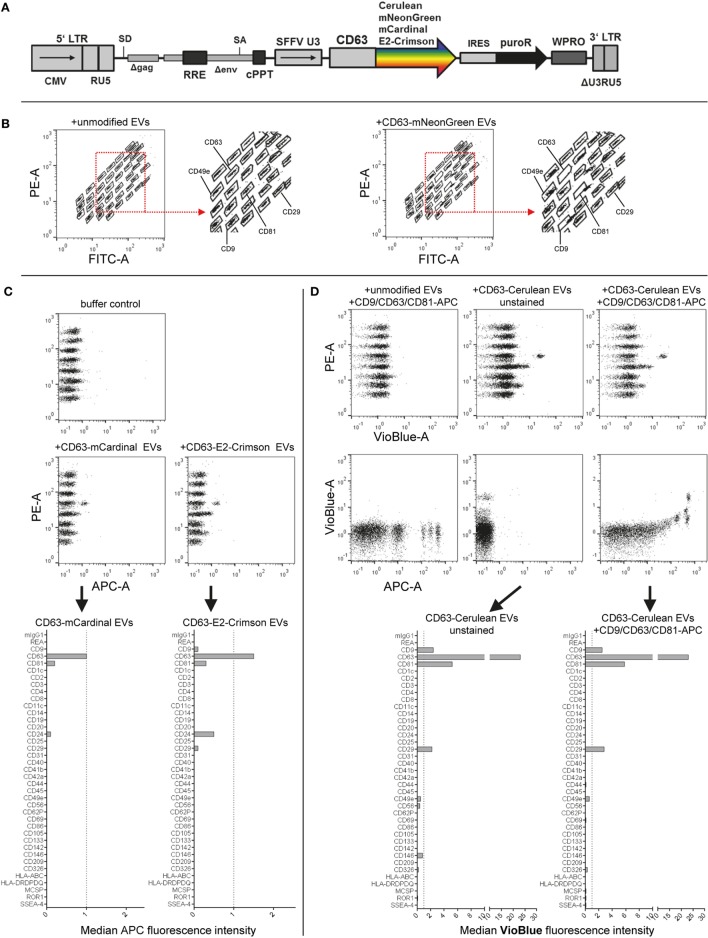
Assay compatibility with fluorescently labeled extracellular vesicles (EVs). **(A)** Schematic outline of the lentiviral vector used. Abbreviations: CMV, CMV promoter; SD, splice donor; LTR, long terminal repeat; SA, splice acceptor; RRE, Rev responsive element; cPPT, central polypurine binding tract; SFFV U3, U3 promoter of the spleen focus forming virus; IRES, internal ribosomal entry site; puroR, puromycin resistance cDNA; WPRO, woodchuck hepatitis virus post-transcriptional regulatory element optimized, modified after Wiek et al. (62). **(B)** Capture bead distribution after overnight incubation with unmodified or CD63-mNeonGreen EVs. **(C)** Capture bead signals in the APC channel after incubation with EVs labeled with far-red fluorescent proteins (mCardinal and E2-Crimson). **(D)** Signals detected in APC and VioBlue channels for unstained and stained (pan tetraspanin) CD63-Cerulean EVs. See Figure S6 and Table S2 in Supplementary Material for further details.

Fluorescent labeling of EVs is often applied to further study EVs, especially in the context of cellular uptake, release or *in vivo* biodistribution. Toward this, we and others have previously labeled EVs with fluorescent proteins by expressing respective CD63 fusion proteins in producer cells (8, 50, 71). Here, we aimed to evaluate the compatibility of EVs labeled with different fluorescent proteins in this assay. Thus, we cloned four different lentiviral constructs facilitating the expression of CD63 fused to either mNeonGreen, mCardinal, E2Crimson, or Cerulean and generated HEK293T cell lines stably expressing each fusion construct (Figure 5A). Labeling of EVs with mNeonGreen, with its fluorescence mainly detected in the FITC channel similar to classical GFP, resulted in increased green fluorescence being detected especially for the CD9, CD63, and CD81 beads and thus interfered with identification of the respective bead populations (Figure 5B; Figure S6C in Supplementary Material). Thus, the use of green fluorescent proteins is not recommended. Labeling of EVs with the far-red fluorescent proteins mCardinal or E2Crimson facilitated detection of some EV surface markers without any further labeling with detection antibodies. Detected signals were generally much lower than those stained with detection antibodies, with slightly higher signals for E2Crimson-labeled EVs than for mCardinal-labeled EVs. The main markers detected comprised CD9, CD63, CD81, CD24, and CD29, which were all confirmed to be present on HEK293T EVs above (Figure 5C; Figures S6D,E in Supplementary Material). While far-red fluorescent proteins will surely not be compatible with the use of APC-conjugated detection antibodies in this assay, they could be of interest as a positive control, reference or to facilitate a more unbiased, non-antigen-dependent detection. The relatively low signals are probably mainly caused by their suboptimal excitation with the equipped 635 nm red laser, since both mCardinal and E2Crimson have an excitation peak around 605 nm and would thus probably be detected at higher signal intensities with a more suitable laser setup (55). Signals from Cerulean-labeled EVs were mainly detected for CD9, CD63, CD81, and CD29 in the VioBlue channel without notable fluorescence spillover from or to the APC channel (Figure 5D; Figures S6F,G in Supplementary Material), suggesting that the use of Cerulean-labeled EVs appears to be fully compatible with this assay in its original form. These results demonstrate that this multiplex bead-based assay facilitates EV surface marker detection with more than one color. This suggests an interesting approach to providing further information about co-expression of EV surface markers in heterogeneous samples. These examples further demonstrate that fluorescently labeled EVs, in this case generated through expression of CD63-fusion constructs in EV producer cells, can be used with the multiplex bead-based assay when appropriate controls are included. This approach should be extended with further validation for lipophilic fluorescent dyes and by applying differently labeled antibodies against different antigens to enhance assay resolution for detection of EV subpopulations.

### Differential EV Surface Marker Detection on EVs Derived From Different Cell Types

Currently, in addition to different EV subtypes being defined based on their origin as exosomes or microvesicles, it is commonly accepted that there is a much higher degree of EV heterogeneity also within these two subgroups. The EV content, including the protein and surface marker composition, is probably strongly dependent on the cell source, the cell’s activation status, and multiple other parameters (5, 7, 9, 10, 72). Since the knowledge about EV subtype and cell source-specific EV surface markers is still rather limited, we next aimed to explore both the use of the multiplex bead-based assay for the analysis of EV heterogeneity within one EV sample, and for the comparison of EVs derived from different cell types.

**Figure 6:**
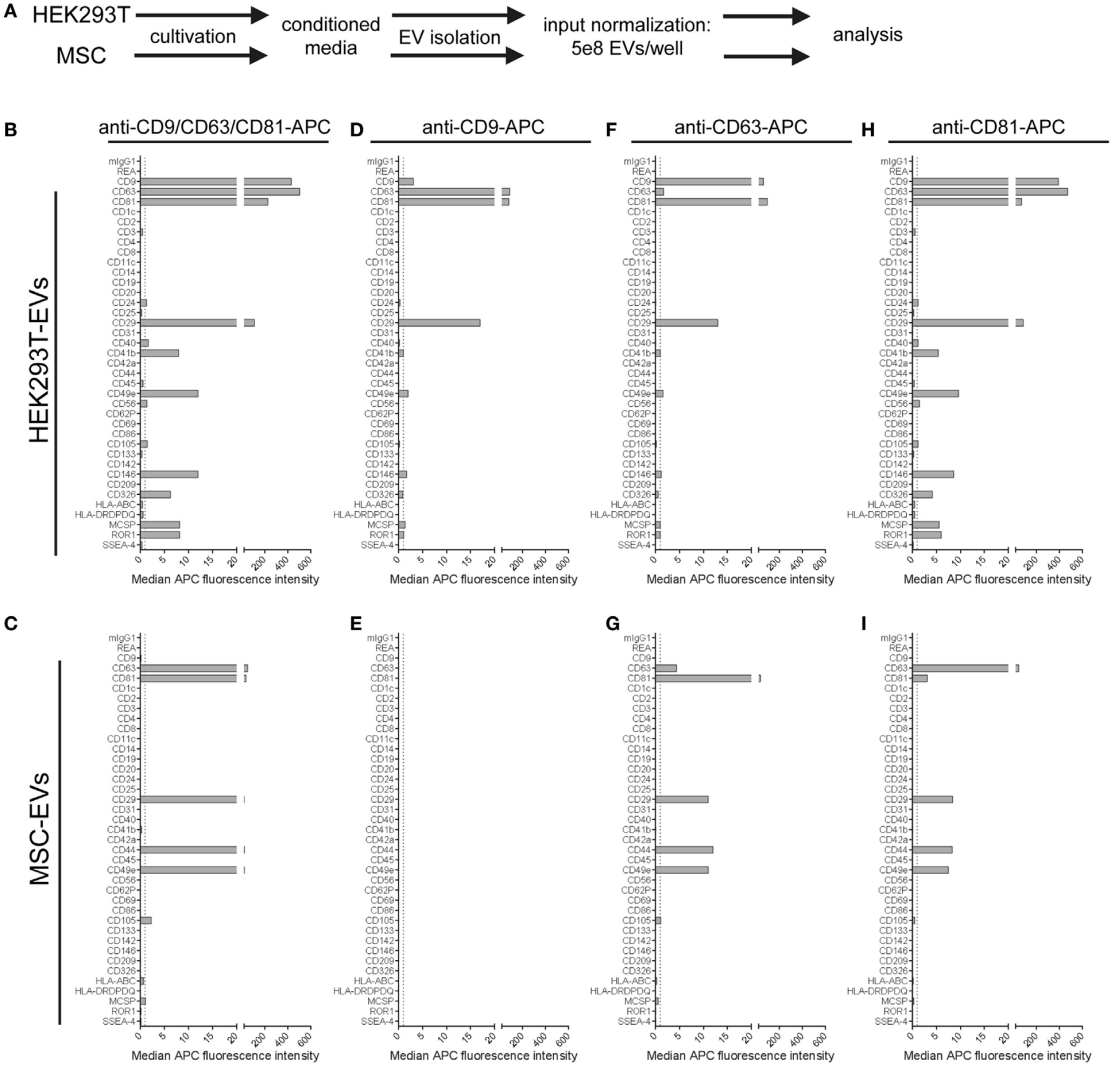
Comparison of extracellular vesicles (EVs) derived from different cell sources. **(A)** Following assay input standardization of 5 × 10^8^ EVs per assay for EVs derived from either HEK293T cells or immortalized hTert + mesenchymal stromal cell lines, different single and pan tetraspanin antibodies were used for detection of EVs captured by capture bead subpopulations. **(B–I)** Representative quantifications of respective EV surface signatures. See Table S2 in Supplementary Material for further details.

Extracellular vesicles were isolated from HEK293T cells and an immortalized MSC line, and input amounts were standardized to 5 × 10^8^ EVs per assay. For detection, either the anti-CD9/CD63/CD81 (pan tetraspanin) detection antibody mix or respective single anti-tetraspanin antibodies were used in order to compare EV surface signatures in detail between both cell sources (Figure 6A). With pan detection, CD63, CD81, CD29, and CD49e were clearly positive on EVs of both cell lines. Of note, the expression of CD9, CD146, CD326, MCSP, and ROR1, was detected on HEK293T EVs but not on MSC-EVs (Figures 6B,C). Subsequently, when using anti-CD9 detection antibodies, we observed a complete lack of signal detection for MSC-EVs, while there was a clear signal on HEK293T EVs (Figures 6D,E). Robust signal detection was observed with anti-CD63 or anti-CD81 detection antibodies on both MSC- and on HEK293T EVs (Figures 6F–I).

We further observed strong variations in signal intensities for the CD9, CD63, and CD81 bead populations when using the same respective antibodies for single detection (Figures 6D,F,H). These variations were most likely caused by limitations in specific antibody epitopes per EV, e.g., signals from EVs that were captured on anti-CD63 beads have less CD63 epitopes available or accessible for detection with anti-CD63 detection antibodies. However, some markers, such as CD29 or CD49e, were detected with all three single detection antibodies on HEK293T EVs (Figures 6D,F,H), and with anti-CD63 and anti-CD81 antibodies on MSC-EVs (Figures 6G,I). On the other hand, CD49e as well as CD146, MCSP, and ROR1 were detected at highest levels on HEK293T EVs when using anti-CD81 antibodies for detection, indicating that those surface markers on HEK293T EVs are co-expressed more frequently on CD81 positive EVs (Figure 6H).

In summary, this dataset shows that the multiplex bead-based assay is suitable to detect heterogeneity within one EV sample, and can also be a valuable tool for comparing EVs from different cell sources. However, a reliable comparison will only be possible if input amounts are standardized to comparable EV input doses, and if EVs are prepared according to similar EV isolation protocols. As discussed above, the absence of signal for a marker might indicate its low abundance under the detection threshold and does not necessarily mean it is not present, as it may just be below the limit of detection for the assay. Although in this case, with CD9 being present at high levels on HEK293T-derived EV samples and not detectable at all on MSC-EVs, even when using anti-CD9 antibodies as a detection probe, it is tempting to conclude that EVs from this particular MSC line are indeed negative for CD9. In fact, EVs derived from a primary MSC line have previously been reported to be positive for CD63 and CD81 but negative for CD9 (73). Western blot analysis confirmed this result, with detectable CD9 signals on HEK293T EVs but not on MSC-EVs (Figure S7 in Supplementary Material). Yet, in this case 5 × 10^9^ HEK293T EVs were required to detect CD9 by WB (Figure S7 in Supplementary Material), while 5 × 10^6^ HEK293T EVs were sufficient to robustly detect CD9 in the multiplex bead-based assay (Figure 2). Thus, the CD9 levels on MSC-EVs from this particular cell line could indeed just be much lower than on HEK293T EVs and below the detection limit of both methods. Of note, CD9 was detected in whole cell lysate of both HEK293T and MSCs (Figure S7 in Supplementary Material). Proteomic profiling of EVs from both HEK293T and this MSC line further did not show any detectable CD9 in MSC-EVs but in HEK293T EVs, while CD63 was detected in both (data not shown, manuscript in preparation). Further validation with other MSC lines, other antibody clones and other, more sensitive methods will be required to further clarify this. CD9 is often referred to as a common EV marker (11, 74–76), but was recently reported to be negative on EVs derived from human primary NK cells, while being highly positive on platelet-derived EVs (10). This further underlines that the tetraspanins CD9, CD63, and CD81 might not be as homogenously distributed on all EVs as previously proposed.

### Usage of Custom Detection Antibodies to Analyze EV Surface Expression

In the default format, this multiplex bead-based assay is used with detection antibodies against single or multiple tetraspanins. Since these are commonly found on most EVs, they can be assumed to be highly abundant when detected in combination. In order to ensure that most EVs are detected, this would also be recommended for experiments aiming to assess the general and complete surface signature of a given EV preparation. However, the question of if a certain candidate surface marker is present on EVs from a given sample will likely be a recurring question in many situations when doing EV research. Thus, we wondered if other markers with potentially lower abundance than tetraspanins would be suitable as detection antibodies in this assay.

**Figure 7:**
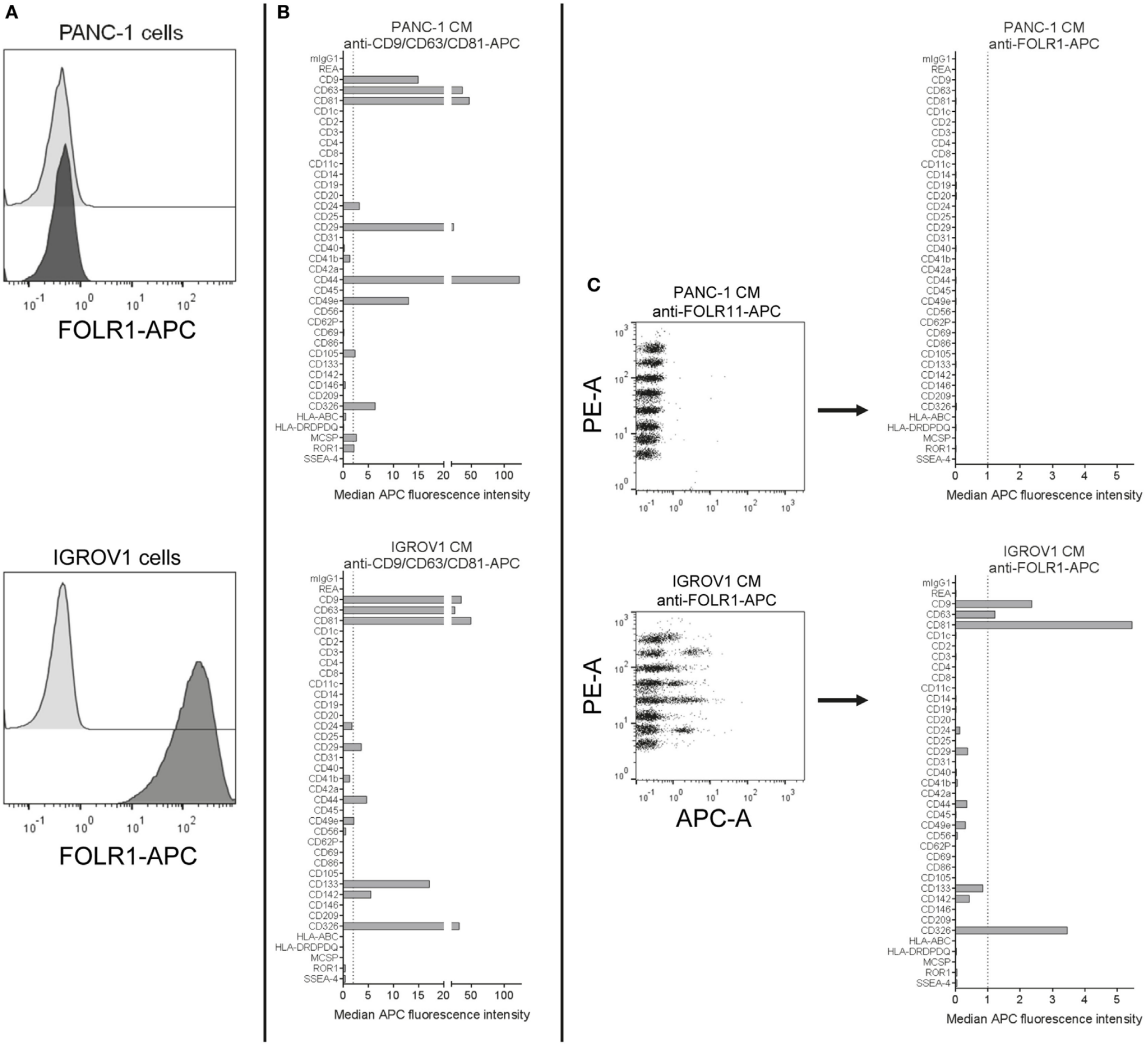
Detection of FOLR1 on human cell culture-derived extracellular vesicles. **(A)** PANC-1 and IGROV1 cells stained with APC-conjugated anti-FOLR1 antibodies (dark gray) compared to respective unstained controls (light gray). **(B)** Conditioned medium (CM) from PANC-1 and IGROV1 cells analyzed in the multiplex bead-based flow cytometric assay, stained with pan tetraspanin-APC detection cocktail. **(C)** The same samples as in **(B)** stained with APC-conjugated anti-FOLR1 antibodies. The figure shows representative data from one out of at least two independent experiments. See Table S2 in Supplementary Material for further details.

The presence of folate receptor alpha (FOLR1) on EVs that shuttle folate into the brain has been reported before (77, 78). Here, we aimed to evaluate the use of anti-FOLR1 antibodies to probe EVs derived from either the pancreatic ductal adenocarcinoma cell line PANC-1, which does not express FOLR1 on its surface, or the ovarian adenocarcinoma cell line IGROV1, which does (Figure 7A). When subjecting pre-cleared CM from both cell lines to the multiplex bead-based assay with pan tetraspanin detection, EVs from both cell lines showed robust expression of abundant markers like tetraspanins, the integrins CD29 and CD49e, and other markers like CD326/EpCAM (Figure 7B). However, when using APC-conjugated anti-FOLR1 antibodies for detection in the same samples, while we did not detect any signals for PANC-1 EVs, we observed clear signals on IGROV1 EVs for the CD9, CD63, CD81, and EpCAM capture bead populations (Figure 7C). This indicates that EVs derived from IGROV1 cells but not PANC-1 cells do express FOLR1 on their surface, and that those EVs co-express tetraspanins and EpCAM. To determine whether further markers are co-expressed, the EV input dose would have to be increased and single anti-tetraspanin antibodies for detection would have to be applied. However, this dataset clearly shows that pre-cleared CM can generally be used to verify if a candidate marker is present on EVs in a given sample, and to gain information on which markers included in the assay are predominantly co-expressed.

In principle, the following requirements for the detection of a candidate antigen or surface marker of interest on the surface of EVs of a given sample will apply: (i) the antigen has to be abundant enough in the tested EV sample to be detected in this assay, (ii) an (ideally APC- or AlexaFluor647-) conjugated antibody is required, (iii) any background signal introduced by that antibody in buffer controls (capture beads + a*ntibody) has to be lower than the true positive signal derived from the sample (capture beads + EVs + antibody), and (iv) the candidate surface marker would have to be co-expressed on the same EVs with at least one of the 37-specific EV surface markers probed for by the capture bead populations, otherwise it would not be picked up.

### Analysis of EV Surface Signatures in Biological Fluids

Due to the potential relevance of EV surface signatures in the context of diagnostic application, their robust and specific assessment in biological fluids, such as CSF, blood, urine, or saliva is of great importance. Even though the focus of this study clearly lies in evaluating the multiplex bead-based assay for the analysis of cell culture-derived EVs, we also wanted to explore the use of this assay to analyze EVs in human CSF, plasma, and serum samples and share our experiences here. Furthermore, we wished to address the question how data from such analyses of larger sets of samples could be visualized, how results from different instruments compared, and how data could be normalized between such sample sets.

**Figure 8:**
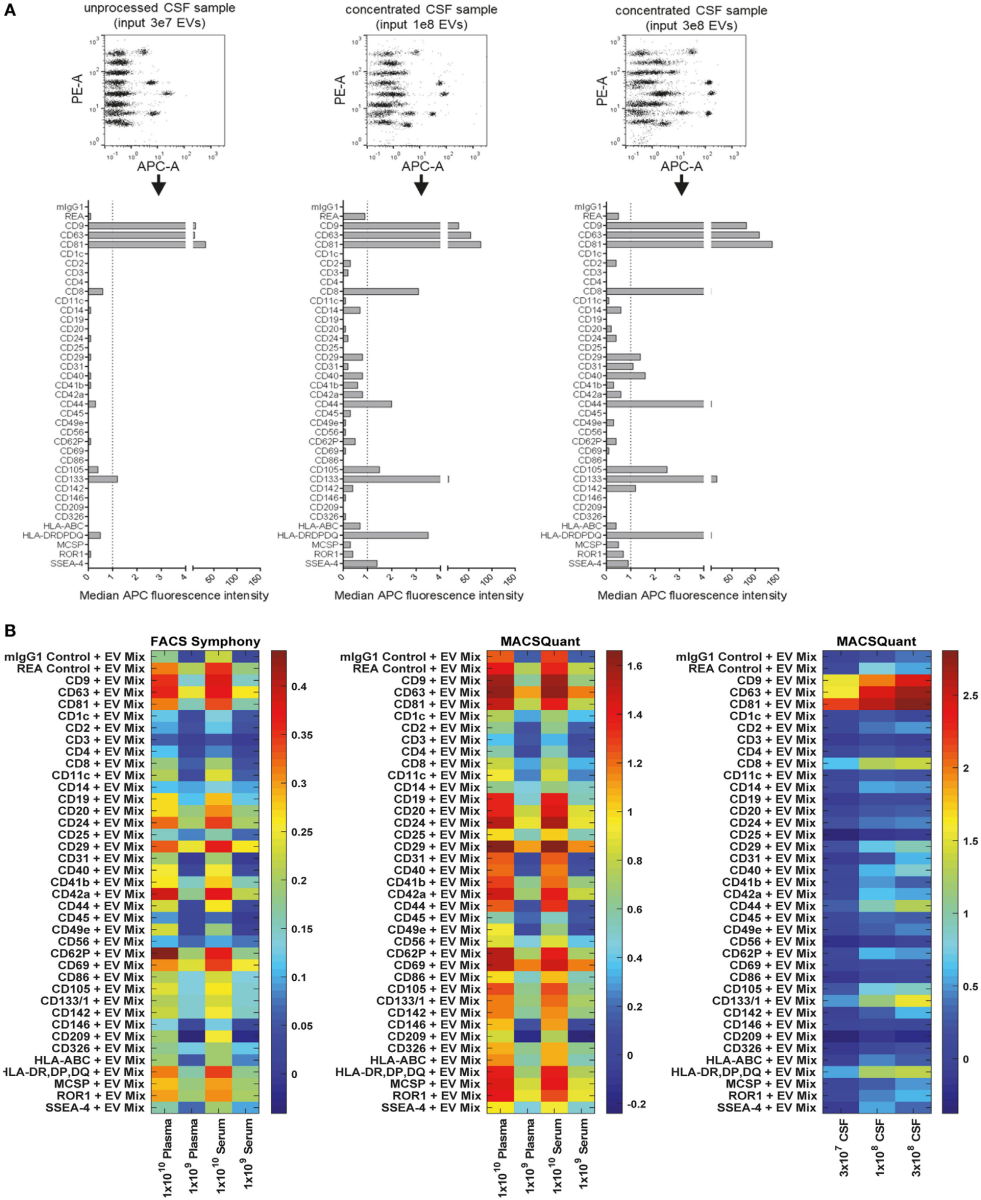
Assessment of extracellular vesicle (EV) surface signatures in human biological fluids. **(A)** Pre-cleared cerebral spinal fluid (CSF) samples measured in three dilutions to study the relevance and specificity of markers with median fluorescence intensity (MFI) close to background levels by observing the MFI increase with increasing EV input amounts. The markers clearly detected as positive in unprocessed CSF (CD9, CD63, CD81, CD133) were consistently detected in all CSF samples analyzed (not shown, eight donors analyzed). **(B)** Heatmap comparison of plasma and serum samples analyzed on the FACS Symphony (left) and MACSQuant (middle) instruments with two different input amounts. A heatmap of the CSF-derived samples from **(A)** is also shown for comparison. Scales are the log10 ratio of APC intensity of the capture beads + extracellular vesicles + antibody (CD9 + CD63 + CD81 = EV Mix) when compared to capture beads + antibody controls. See Figures S8–S12 in Supplementary Material for further information and Tables S2 and S3 in Supplementary Material for further details.

When analyzing pre-cleared CSF samples, we consistently observed expression of CD9, CD63, and CD81 and CD133/Prominin1 in samples from all donors. However, other markers were detected consistently in all samples but were near background levels, i.e., CD8, CD14, CD31, CD41b, CD44, CD105, and MHC class II (HLA-DRDPDQ) (Figure 8A, data not shown). This probably related to relatively low EV concentrations in CSF compared to cell culture supernatant (data not shown). To gain more information on whether markers with lower abundance in CSF are specifically expressed, we further concentrated EVs from CSF samples and used approximately 3- and 10-fold more EVs as assay input. Most markers expressed at low levels showed higher intensities in more concentrated samples (e.g., CD8, CD31, CD44, CD105, and MHC class II), while some markers, i.e., CD3, CD14, CD41b, or CD62P did not show any consistent signal increase with increased input doses (Figure 8A). This indicates that interpretation of EV surface markers close to background levels generally requires further validation by running samples at different dilutions. We further investigated the stability of EV surface signatures when analyzing pre-cleared, unprocessed CSF samples, and observed reproducible results for the same CSF sample analyzed freshly or after storage at −20°C for up to 2 months (Figure S8 in Supplementary Material), indicating that freezing does not have a major impact on the EV surface signature of CSF samples as detected by this multiplex bead-based assay.

Next, we compared different basic sample-related parameters by analyzing human blood-derived samples, i.e., plasma and serum, at different input amounts (10^8^–10^10^/assay), different EV purities and fresh Vs. frozen samples. Initial experiments were performed on a FACS Symphony instrument (Figure S9A in Supplementary Material), and the pan tetraspanin-APC intensities for each bead population were visualized on heatmaps (Figure 8B; Figure S10 in Supplementary Material). When comparing non-purified plasma samples with samples purified by SEC, we observed much higher pan tetraspanin detection signals in non-purified samples, though signals were also elevated for the internal negative control bead populations, especially for the REA-control bead population (Figures S8B and S10 in Supplementary Material). This indicates that, compared to experiments with cell culture-derived EV samples, analysis of biological samples with this assay requires optimized sample purification and further validation, since background or unspecific signals can occur. Furthermore, we did not observe any consistent difference between plasma and serum samples (Figure 8B; Figures S10 and S11 in Supplementary Material), or, as observed for CSF, between plasma and serum samples subjected to a freeze-thaw cycle, indicating that EV surface markers are not notably affected by freezing plasma or serum (Figure S10 in Supplementary Material).

For further comparison and validation, the same frozen plasma and serum samples were analyzed using the MACSQuant instrument with the same defined input amounts, and we observed highly comparable results. Results from CSF samples were included in the corresponding heatmaps for further comparison (Figure 8B; Figures S11 and S12 in Supplementary Material). Normalization of results from both instruments enabled us to conclude that the plasma and serum samples analyzed on both instruments showed clear similarity in the markers detected as positive, particularly when comparing plasma and serum samples at an input dose of 1 × 10^9^ EVs. Results from the MACSQuant showed a greater degree of variation in terms of scale, but less variation than observed in expression when compared to the FACS Symphony (Figure 8B; Figure S12 in Supplementary Material). In summary, efforts were taken to minimize variables that could potentially introduce variation, but many are unavoidable due to sample, antibody, and bead handling and preparation, or instrument design differences. While this multiplex bead-based assay is very useful for understanding the general repertoire of EV surface marker expression, it is by no means a completely quantitative analysis technique and, therefore, has limitations that should be understood in order to draw appropriate conclusions from the obtained results. Comparisons between the FACS Symphony and MACSQuant instruments were made possibly by using the ratio of control to EV-captured beads, rather than using background subtraction, due to lack of positive controls. Future inter-instrument comparisons may benefit by converting arbitrary unit scales to molecules of equivalent soluble fluorophore (MESF) using MESF beads to account for instrumental sensitivity and scaling differences that can arise.

Taken together, in terms of detected EV surface signatures in CSF Vs. blood-derived samples, further validations with additional samples will be required in order to make clear statements about which markers are generally positive on EVs from most donors and which markers are more variable. CSF samples contained lower EV concentrations and much lower background levels than plasma/serum samples. CD9, CD63, and CD81 expression was observed at high levels for both EVs from CSF and from blood. Further markers with high abundance on CSF EVs were the T cell marker CD8, MHC class II, and CD133, with especially CD8 and CD133 being much higher on CSF-EVs than blood EVs (Figures 8A,B; Figure S11 in Supplementary Material). The presence of MHC-II on CSF-EVs was reported before (79), and expression of the stem/progenitor cell marker CD133/Prominin1 on EVs in CSF was first described in 2008 (80), with a more recent study proposing its dose-related association with several neurological diseases (81). Due to relatively high signals observed for internal negative control bead populations for plasma/serum EVs, at this point it is difficult to identify surface markers unequivocally positive on blood EVs with this multiplex bead-based assay in its current form. It appears that especially the REA control capture bead population which is coated with a recombinant isotype control antibody against keyhole limpet hemocyanin somehow binds to molecules present in plasma/serum, but not in CSF or cell culture supernatant. However, the highest signal intensities above background levels for plasma/serum samples were obtained for CD24, CD29, CD42a, CD62P, and CD69 (Figure 8B; Figure S11 in Supplementary Material). In general, further optimization and analysis of more samples from defined donor groups will be required to explore potential disease associations of specific EV surface marker combinations.

### Detection of Human EVs in Mouse Plasma Following Intravenous Injection

Within the past few years, there have been rapidly accumulating reports about the therapeutic potential of EVs, and especially of MSC-derived EVs. Treatment with MSC-EVs has been reported as being beneficial and safe in several animal studies, and also in the first case in man (82–85). However, the mode of action of EVs in such therapeutic settings is still poorly understood, in part because it is technically challenging to track the biodistribution of EVs over time. Different labeling techniques, e.g., labeling of EVs with lipophilic dyes, fluorescent proteins, or luciferase-based approaches have been applied to study the biodistribution of EVs injected in mouse models over time (8, 67, 86, 87). Several of these studies have shown that following injection, EVs are rapidly cleared from the blood circulation and mostly accumulate in liver and lungs. However, each labeling technique is associated with different limitations, such as the risk of merely tracing the dye or fluorophore. The use of chimeric proteins with a luminescent or fluorescent tag fused to an EV sorting moiety has the advantage of high specificity and lowered risk of signal from non-EV elements. However, this specificity may also be disadvantageous, since it will only reflect the EV population carrying the respective EV sorting domain. Thus, since it is challenging to follow EVs over time, or to detect if specific EV subsets behave differently in terms of biodistribution or targeting after injection, any assay that can be used to gain additional information in such mouse models will be helpful to further understand EV heterogeneity, biodistribution, targeting, and function.

**Figure 9:**
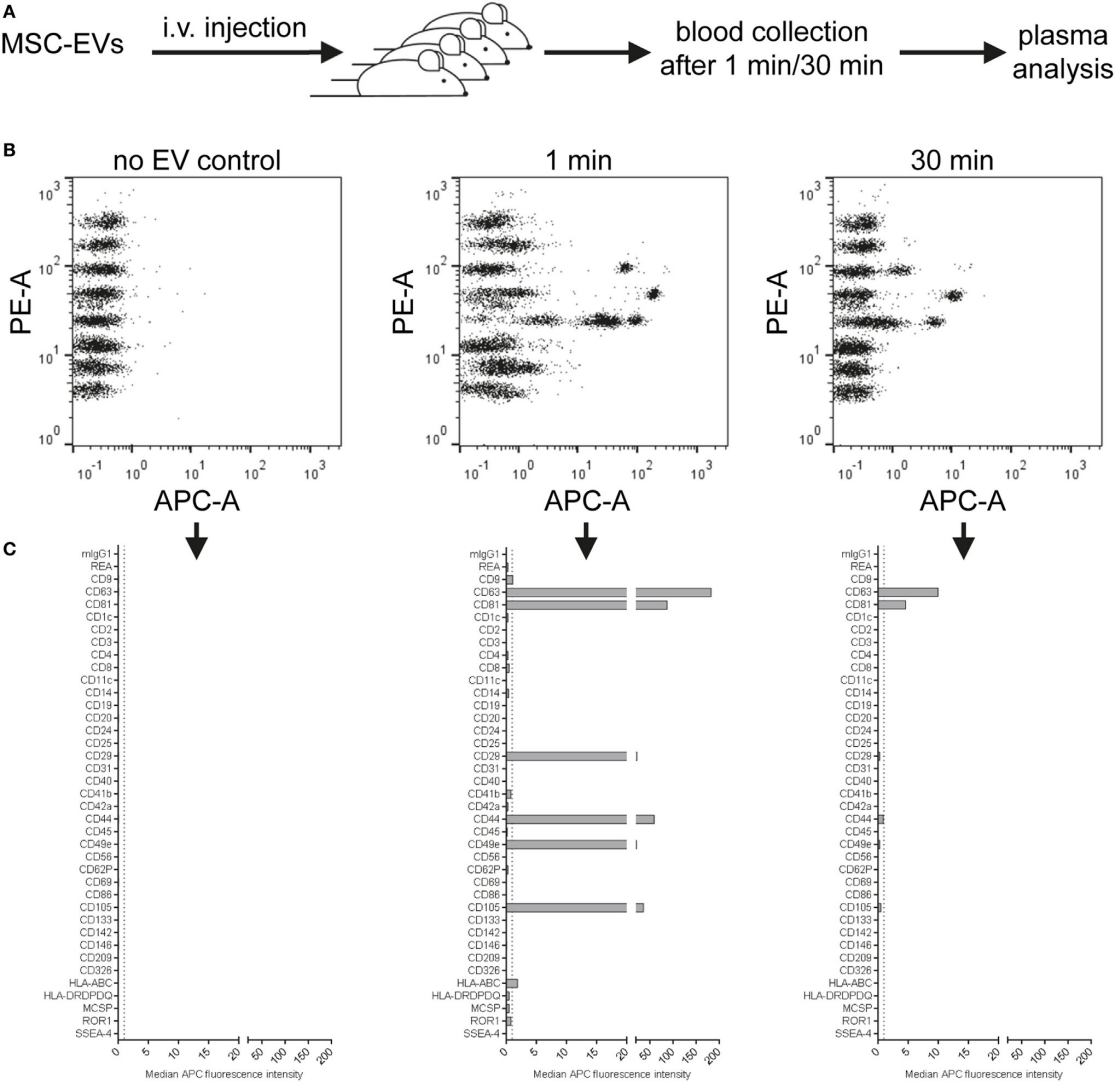

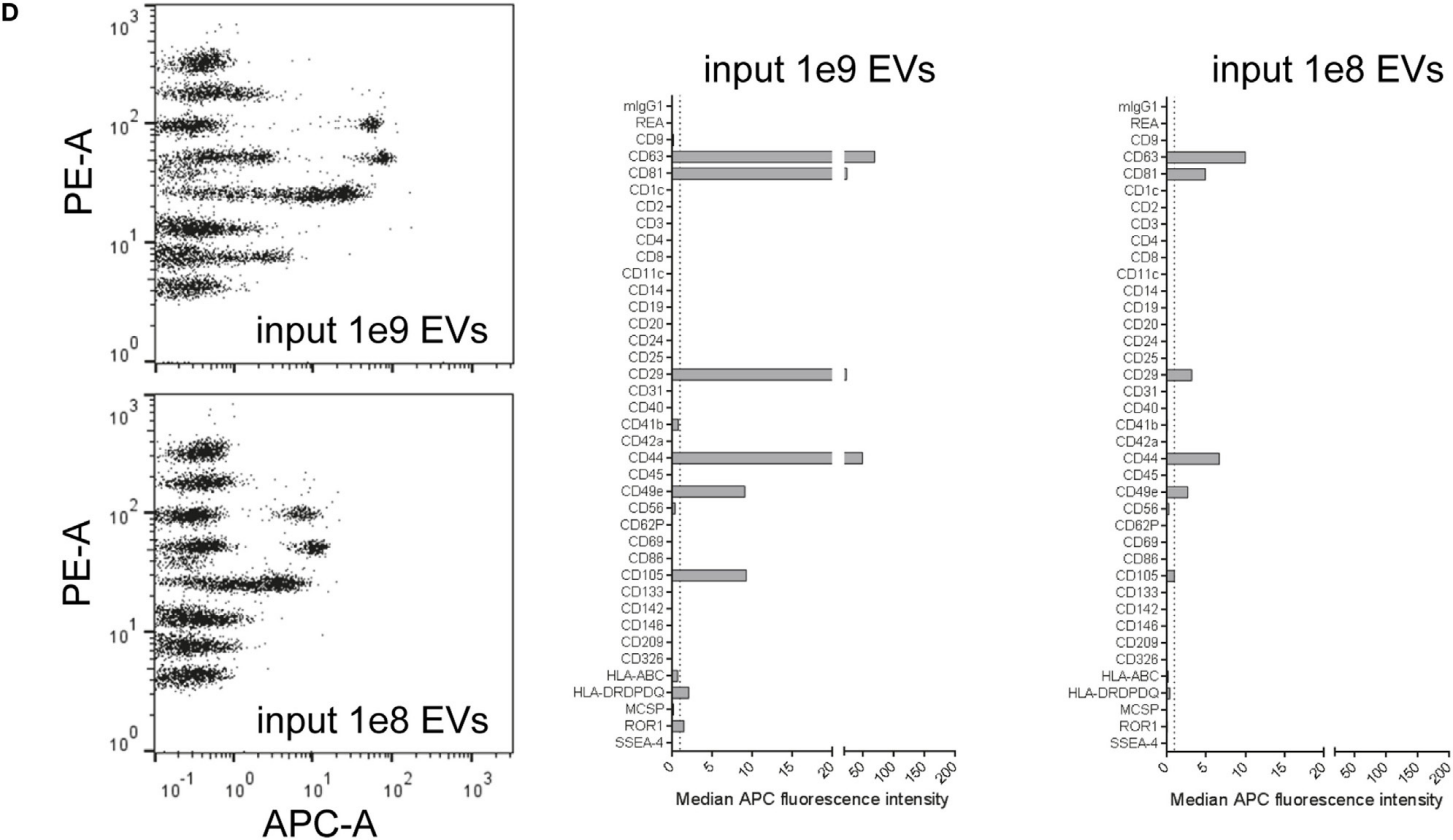
Detection of injected human extracellular vesicles (EVs) in mouse plasma. **(A)** Female NMRI mice were injected intravenously at doses of 2 × 10^11^ human mesenchymal stromal cell line-EVs/mouse. Blood was collected 1 or 30 min post injection by heart puncture. Plasma from two to three mice for each time point was analyzed in the multiplex flow-cytometry assay by using a pan tetraspanin mix of CD9/CD63/CD81-APC antibodies. **(B)** Representative dot plots and **(C)** quantification of detected bead populations from analyzed plasma samples from both time points compared to a plasma sample from a non-injected mouse. **(D)** The same EV preparation was analyzed directly at assay input doses of 10^9^ and 10^8^ EVs for comparison. See Figure S13 and Table S2 in Supplementary Material for further details.

Since this multiplex assay should be rather specific for detection of human EVs, we hypothesized that it could also be useful for the analysis of non-manipulated, human EVs after injection into a mouse. Thus, we isolated EVs from an immortalized human MSC line, injected doses of 2 × 10^11^ EVs/mouse intravenously and took blood samples after 1 min, 30 min, and later time points (Figure 9A, data not shown). Plasma samples from these mice were then transferred without further dilution to the multiplex bead-based assay with pan tetraspanin detection. We did not observe any signals from plasma samples from non-injected mice, but could observe clear EV detection after 1 min and lower signals 30 min after injection of treated mice (Figure 9B). The surface markers detected accurately 1 min after injection (Figure 9C) reflected the surface markers present on MSC-EVs that were directly analyzed at different doses (Figure 9D). Plasma samples taken 30 min after injection showed drastically reduced signals (Figures 9C,D) and were below the limit of detection for this assay 1 h after detection and at later time points (data not shown). In this experiment, we also tried to use the exact injection dose of 2 × 10^11^ EVs as input dose for the multiplex bead-based assay for comparison, without taking the dilution of EVs in mouse blood into account; however, this resulted in massive background signals also for the internal negative control bead populations (Figure S13 in Supplementary Material).

Taken together, this proof-of-concept experiment shows that this multiplex bead-based assay can facilitate the specific detection of native, non-manipulated human EVs, and their surface signatures from the blood of EV-injected mice. In accordance with previous findings, we observed that most EVs are cleared from the circulation rapidly. Though, this approach requires further optimization in order to gain deeper insight into which tissues different subsets of EVs are targeted to. Such data would greatly complement other biodistribution or pharmacokinetic studies, which normally rely on bulk fluorescence or luminescence signals and fail to assess surface signatures. Furthermore, this approach could potentially be a powerful tool to detect otherwise non-manipulated EVs in stem cell or cancer xenograft studies.

### Detection of EV Surface Signatures in Material Derived From Rare Primary Human Hematopoietic Progenitor Subsets

As in many other fields, a role for EVs in intercellular communication has also been proposed in context of communication between hematopoietic stem and progenitor cells (HSPCs) and bone marrow niche cells or leukemia cells. The disruption of this communication axis has been hypothesized to contribute to leukemia development (88–90). Since a general understanding of EV subtypes and the identification of specific EV surface markers will be essential to studying and understanding cell-to-cell communication processes within the hematopoietic compartment, we aimed to evaluate if this multiplex bead-based assay would be sensitive enough to assess EV surface signatures from subsets of rare cells like HSPCs, with low total cell numbers and low supernatant volumes available.

**Figure 10:**
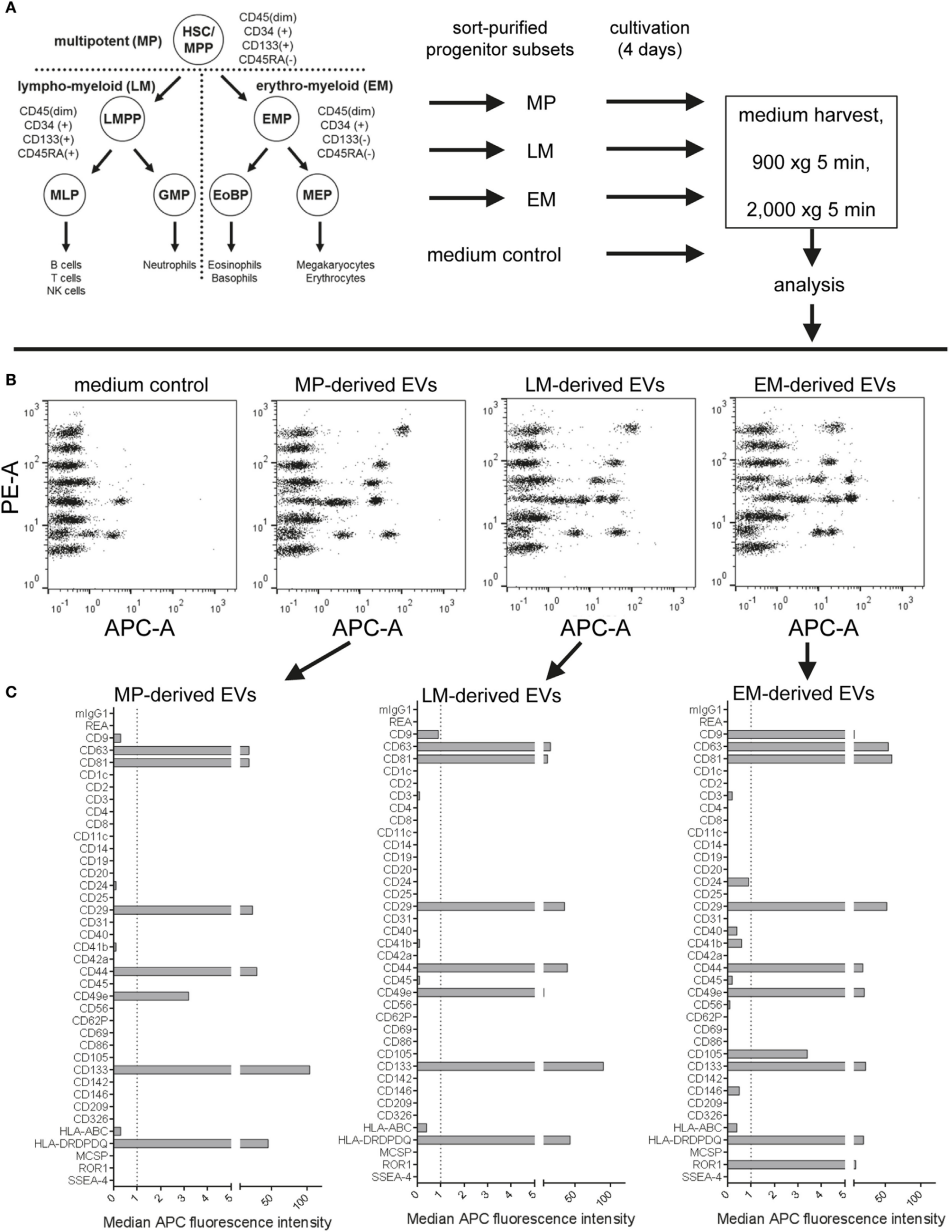

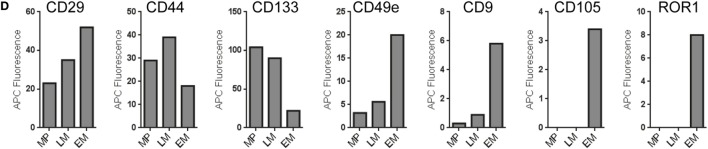
Analysis of extracellular vesicles (EVs) secreted by subsets of human hematopoietic stem/progenitor cells. **(A)** Human umbilical cord blood-derived hematopoietic stem and progenitor cell (HSPC) subtypes were purified *via* flow cytometric cell sorting, seeded in 48-well plate at doses of 25,000 cells per well in 300 µL medium and cultured for 4 days before supernatants were harvested, pre-cleared by centrifugation, and analyzed in the multiplex bead-based assay. Medium controls were included for all conditions and treated exactly like samples. **(B)** Dot plots showing raw data for medium control and supernatants from multipotent (MP), lympho-myeloid (LM), and erythromyeloid (EM) HSPC subset-derived supernatants. **(C)** EV surface signatures for EVs from the three HSPC subsets, background corrected by subtraction of medium control. **(D)** Expression of selected markers detected in conditioned medium derived from the HSPC subsets. Data from one out of two independent experiments are shown. See Figure S14 and Table S2 in Supplementary Material for further details.

To analyze if subsets of hematopoietic progenitor cells secrete different qualities or classes of EVs, we sort-purified HSPC fractions known to be enriched for multipotent (MP), lympho-myeloid (LM), and erythromyeloid (EM) progenitor cells (52) (Figure 10A; Figures S14A,B in Supplementary Material) and cultured these fractions at doses of 25,000 cells per 48 well in 300 µL medium for 4 days, respectively. All CM were not filtered at time of harvesting due to low volumes, but cleared from cells and debris/large vesicles by low speed centrifugation before analysis with pan tetraspanin detection (Figure 10A; Table S2 in Supplementary Material). Medium controls were included and treated exactly like samples (Figure 10A). The medium controls showed considerable signals which were corrected for by using the respective MFI values from this control for background subtraction (Figure 10B). Generally, the signals derived from all samples were high enough above background to facilitate the identification of several markers as clearly expressed at differential levels on MP-, LM-, and EM-derived EVs (Figures 10B,C). While some markers like CD29 and CD44 could be identified at rather high and similar levels on EVs derived from all three subsets, several markers were found at clearly higher levels or were detected exclusively on EM-derived EVs, e.g., CD9, CD49e, CD105, and ROR1. On the other hand, the marker CD133 was found to be consistently higher on MP- and LM-derived EVs (Figures 10C,D; Figures S14C,D in Supplementary Material), which is likely due to the fact that the MP and LM HSPC EVs were derived from cells expressing high levels of CD133 on their surface, while EM progenitors express CD133 at much lower levels (Figure S14B in Supplementary Material) (52, 58). When comparing the results from two independent experiments, all trends in EV surface marker expression level variation between MP, LM, and EM-derived EVs were highly similar (Figures S14C,D in Supplementary Material).

Taken together, this dataset shows that this multiplex bead-based assay facilitates the analysis of experiments from limited cellular material and low supernatant volumes with high reproducibility. We can further conclude that this assay enables the detection of both similar and also consistently differential expressed surface markers on EVs from different HSPC subsets, which in turn indicates that the overall surface signature of EVs derived from all three subsets is different. Finally, further optimization and more dedicated single vesicle analysis will be required to properly validate differences detected between EVs from these HSPC subtypes.

### Concluding Summary and Outlook

In summary, we have comprehensively evaluated and optimized a multiplex bead-based flow cytometric assay that can be used to robustly detect EV surface signatures in a semi-quantitative way. More specifically, the results presented in this study demonstrate that this assay facilitates EV surface marker detection in different types of samples in a very specific and reproducible way. To our knowledge, this is the first study that comprehensively evaluates the methodological details of a flow cytometric method for EV surface marker detection that does not need dedicated instrumentation or extensive operator expertise. Of note, the specificity is primarily based on the need for two antibodies to bind one EV in order to detect any signal, which makes this assay both biased and much more specific and sensitive compared to other classical bulk-based methods to detect EV proteins, such as Western blot. We believe that the EV field will benefit greatly from these kinds of assays and thus we share our experiences here, and give recommendations about sample- and acquisition-related parameters and required controls, which in the end will depend on the type of scientific questions asked. Of note, as with most analytic methods within the EV field, this assay should be considered as complement to other analytic EV tools, e.g., NTA, in order to draw valid conclusions. We demonstrate potential applications for this assay to include the comparison of isolation methods, as an in-process monitoring tool, for the identification of EV surface markers in a given sample and comparison of different samples, as an additional read-out for *in vivo* pharmacokinetics and biodistribution experiments, and for the assessment of EVs in samples with low volume or low EV counts.

In general, the detection of EV surface signatures on cell culture-derived EVs appears to be specific and reproducible. In this study, a few markers were detected robustly on EVs derived from all cell lines (CD63, CD81, CD29, CD49e), and several other markers were detected at least on one cell line tested. Many capture bead populations included in this assay are coated with antibodies against specific blood cell-related antigens and did not show detectable signals for cell culture-derived EVs here. Of note, the specificity of EV binding to the CD9, CD63, and CD81 capture beads has been previously evaluated by antibody blocking experiments (10), and most capture bead populations have been detected as positive in EV samples derived from respective blood cell subsets (e.g., CD19 and CD20 detection for B cell EVs, CD2, CD8, and CD56 for NK cell EVs, and CD42a, CD61, and CD62P for platelet-derived EVs) before (9, 10). Still, future studies should aim to further validate the specificity of other antibodies included in this assay, e.g., by analyzing further cell sources or by blocking experiments. In addition, the analysis of biological fluids, at least of blood-derived EVs, requires further technical optimization and clarification. For example, it remains unclear why the internal control bead populations react with plasma/serum EVs. However, we feel the overall potential to use this robust, multiplexed bead-based flow cytometric assay to generate a snapshot of EV surface signatures in clinically relevant samples with 37-specific markers will be of considerable interest, especially after additional disease-specific EV-associated biomarkers have been identified.

Furthermore, the data obtained in this study clearly underlines that this multiplex bead-based EV flow cytometry kit can not only quantify robust EV surface signatures in a given sample but is also useful for comparing differentially expressed surface markers between samples. It thereby facilitates the identification of heterogeneities between different EV sources, which may lead to the identification of EV markers being specific for certain cell types. The combination of this rather robust and fast approach with more dedicated methods to validate candidate surface markers distinguishing EV subpopulations (i.e., single EV flow cytometric analysis or sorting) would pave the way to studying the function of EV subsets, which will be of the highest relevance to furthering our understanding of their molecular content and related functions.

Future optimization of such an assay and application within the field should aim to identify new EV surface markers, but especially also of validated detection antibodies, more suitable reference materials for controlling experimental parameters and background, and further exploration of the limit of detection in terms of absolute molecules per capture bead needed on a respective instrument to show signals above backgrounds levels for a certain sample type. Finally, the validation of additional fluorescent probes that can be applied in such assays alone or in combination, e.g., the addition of two or more differently labeled detection antibodies to one sample, will add further depth to our knowledge of marker co-expression.

## Ethics Statement

Human UCB was obtained from donors at the University Hospital Essen, Germany, after informed written consent according to the Declaration of Helsinki. The experimental usage of UCB samples was approved by the local ethics commission. CSF samples included in this study were derived from patients who underwent a lumbar puncture for clinical purposes at Neurology department at Karolinska University Hospital, Stockholm Huddinge, Sweden. Written informed consent was obtained from all subjects in accordance with the Declaration of Helsinki. This study was approved by the Regional Ethical Review Board in Stockholm, Sweden. Human blood samples: the prospective clinical studies 02-C-0064, 04-C-0257, and 09-C-0195 were approved by the Institutional Review Board of the National Cancer Institute (NCI; MD, USA). Informed consent was obtained from all donors. The animal experiments were approved by the Swedish Local Board for Laboratory Animals. The experiments were performed in accordance with the ethical permissions granted, and designed to minimize the suffering and pain of the animals.

## Author Contributions

OW, SEA, and AG conceived the idea and designed the experiments. AG wrote the manuscript and assembled the figures. OW, RB, JW, AZ, DH, GC, BG, JN, and SEA assisted in manuscript writing. OW, RB, JW, AZ, FM, UF, BE, X-ML, GC, MG, DM, CW, MBr, DG, and AG cloned plasmids, generated cell lines, performed flow cytometric cell sorting, cultured cells, and/or isolated EVs. OW, UF, and DG performed the *in vivo* experiments. BE isolated the CSF samples. AG acquired most flow cytometric EV analysis data and performed most of the data analysis. JW and JJ acquired flow cytometric data and analyzed data from biological fluid samples. GC performed Western blot analysis. OW, RB, JW, AZ, FM, UF, DH, BE, X-ML, GC, MBr, DG, BG, JN, JJ, SEA, and AG contributed general intellectual input on the experimental design, contributed to data analysis, and/or discussed the results. BE, HH, MBj, and BG contributed to relevant materials and expertise. All authors reviewed the manuscript and approved its final version.

## Conflict of Interest Statement

OW, JN, SA, and AG are consultants for and have equity interests in Evox Therapeutics Ltd. All other authors declare that no potential conflict of interest exists.
