# Supplementary material for: Systematic Methodological Evaluation of a Multiplex Bead-Based Flow Cytometry Assay for Detection of Extracellular Vesicle Surface Signatures
Source: Front Immunol. 2018 Jun 13;9:1326. doi: 10.3389/fimmu.2018.01326 (PMC6008374; doi:10.3389/fimmu.2018.01326)
Supplement: Supplementary file 1 [file Data_Sheet_1.docx]

# Supplementary Information

**Systematic methodological evaluation of a multiplex bead-based flow cytometry assay for detection of extracellular vesicle surface signatures**

Oscar P. B. Wiklander^1;2;#^, R. Beklem Bostancioglu^1;#^, Joshua A. Welsh^3^, Antje M. Zickler^1;4^, Florian Murke^5^, Giulia Corso^1^, Ulrika Felldin^1^, Daniel W. Hagey^1^, Björn Evertsson^6^, Xiu-Ming Liang^1^, Manuela O. Gustafsson^1^, Dara K. Mohammad^1;7^, Constanze Wiek^8^, Helmut Hanenberg^8;9^, Michel Bremer^5^, Dhanu Gupta^1^, Mikael Björnstedt^4^, Bernd Giebel^5^, Joel Z. Nordin^1;2^, Jennifer C. Jones^3^, Samir EL Andaloussi^1;2;10;^ **^ǂ^**, André Görgens^1;2;5;^ **^ǂ^**^;*^

^1^Clinical Research Center, Department of Laboratory Medicine, Karolinska Institutet, Stockholm, Sweden
^2^Evox Therapeutics Limited, Oxford, United Kingdom
^3^Molecular Immunogenetics and Vaccine Research Section, Vaccine Branch, CCR, NCI, NIH, Bethesda, MD, USA
^4^Division of Pathology F56, Department of Laboratory Medicine, Karolinska Institutet, Karolinska University Hospital Huddinge, Stockholm, Sweden
^5^Institute for Transfusion Medicine, University Hospital Essen, University of Duisburg-Essen, Essen, Germany
^6^Department of Clinical Neuroscience, Karolinska Institutet, Karolinska University Hospital, Stockholm, Sweden
^7^Department of Biology, College of Science, Salahaddin University-Erbil, Erbil, Kurdistan Region, Iraq
^8^Department of Otorhinolaryngology & Head/Neck Surgery, University Hospital Düsseldorf, Heinrich Heine University, Düsseldorf, Germany
^9^Department of Pediatrics III, University Children's Hospital Essen, University Duisburg-Essen, Essen, Germany
^10^Department of Physiology, Anatomy and Genetics, University of Oxford, Oxford, United Kingdom

**#/ǂ** authors contributed equally to this manuscript

*** Correspondence:**André Görgens
[Andre.Gorgens@ki.se](mailto:Andre.Gorgens@ki.se)

# Supplementary Figures

# Figure S1 (related to Figure 1)


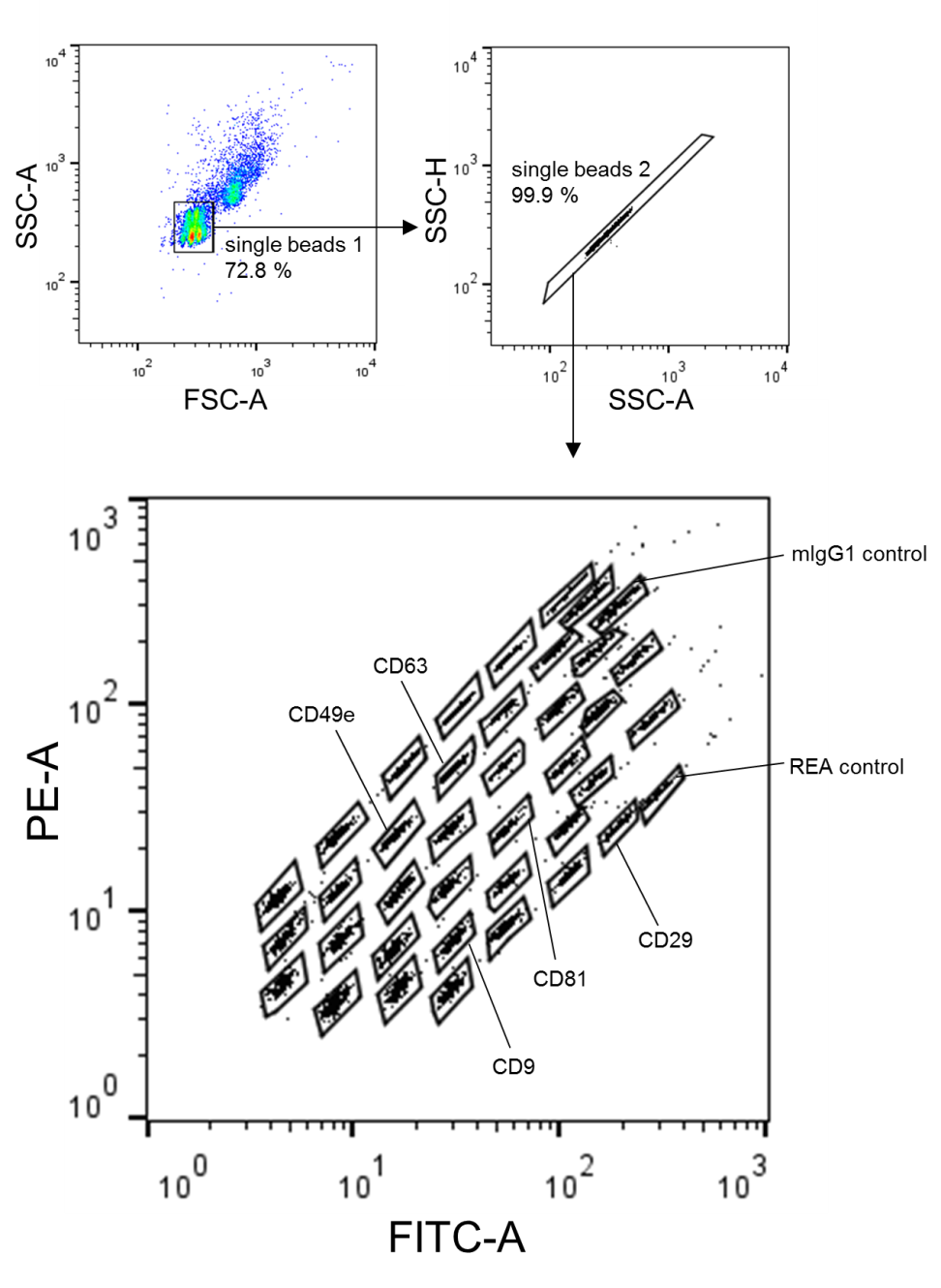


**Figure S1: Gating strategy and examples for the identification of capture bead populations.** The gating strategy applied for gating on single beads, and subsequent gates applied to identify all 39 distinct capture bead populations, each coated with specific monoclonal antibodies, are shown. Labeled bead populations (CD9, CD29, CD49e, CD63, CD81, REA control and mIgG1 control) are given as examples. All bead populations were identified according to the manufacturer’s recommendations.

**Figure S2 (related to Figure 1)**

**
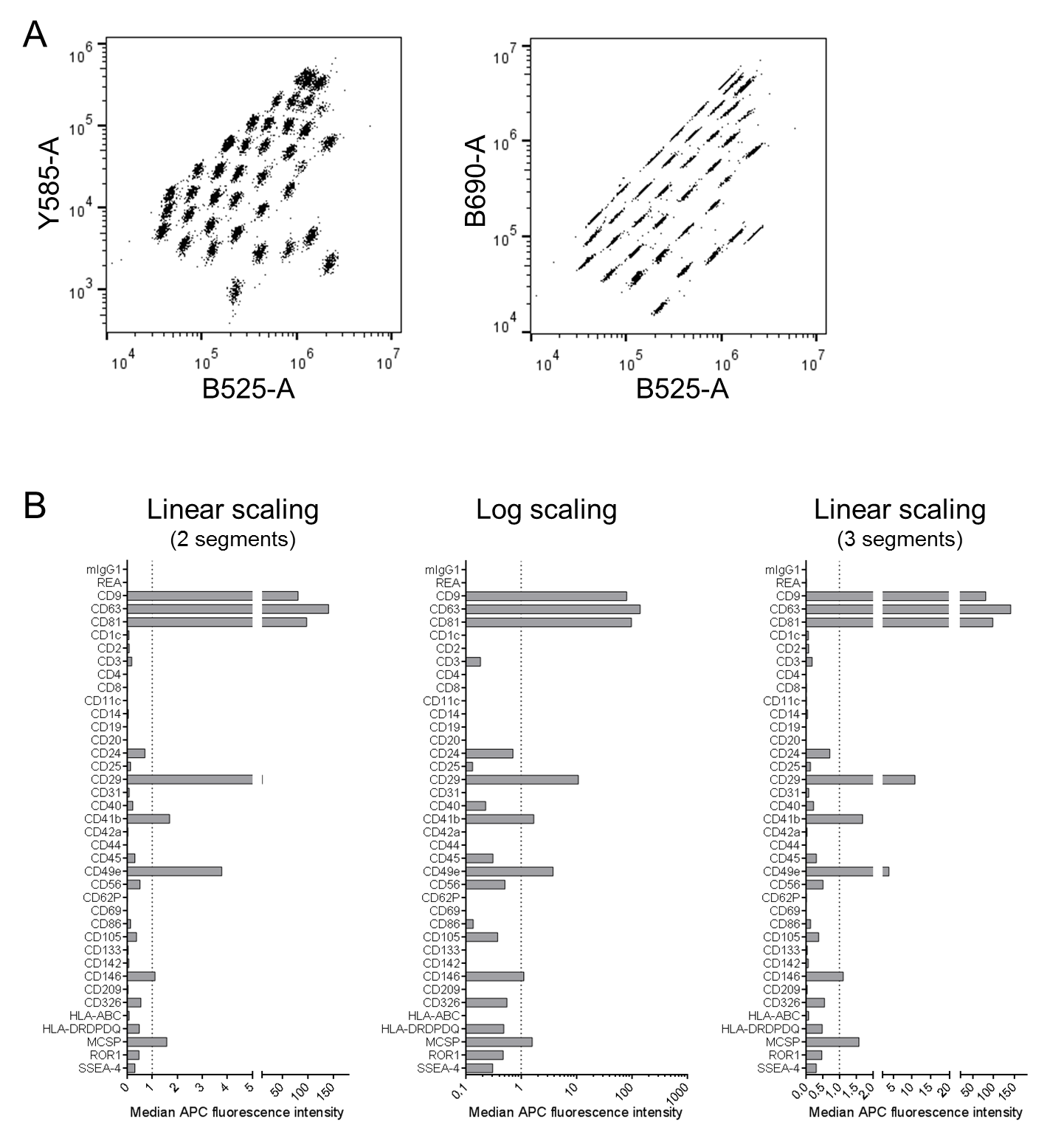
**

**Figure S2:**  **Instrument-dependent bead identification and examples for data presentation.** (A) Examples for differing distribution of bead population on a Cytoflex S instrument equipped with a green excitation laser when using the default filters to detect FITC (B525) and PE (Y585). The distribution of beads is more comparable with data from cytometers without green laser when using appropriate other combinations of filters for signal detection, in this case B525 versus B690 filters. (B) The median APC fluorescence intensities for the same dataset as shown in Figure 1C was plotted by applying three modes of axis scaling, demonstrating how the axis scaling can change especially the visualization of signals derived from markers showing low signals.

**Figure S3 (related to Figure 3)**


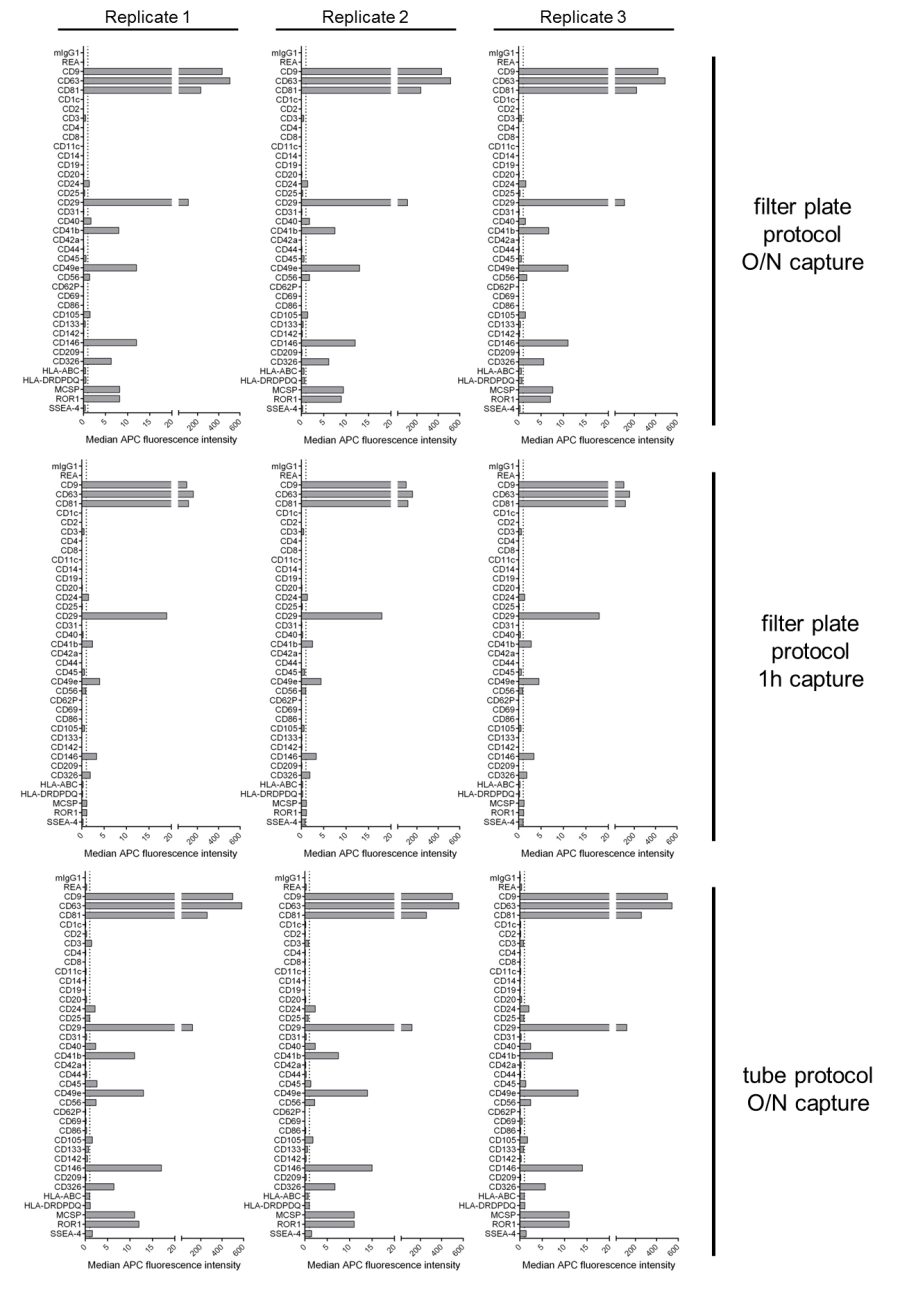


**Figure S3:**  **Sample-to-Sample variability in different protocols.** Complete surface profiles of the experiment shown in Figure 4, with EV surface profiles of three replicates shown for three different protocols: The default protocol used throughout this study (filter plate protocol, O/N capture; top), a protocol with the capture time shortened to 1 hour (middle) and a protocol performed in microcentrifuge tubes instead of filter plates (bottom).

**Figure S4 (related to Figure 3):**


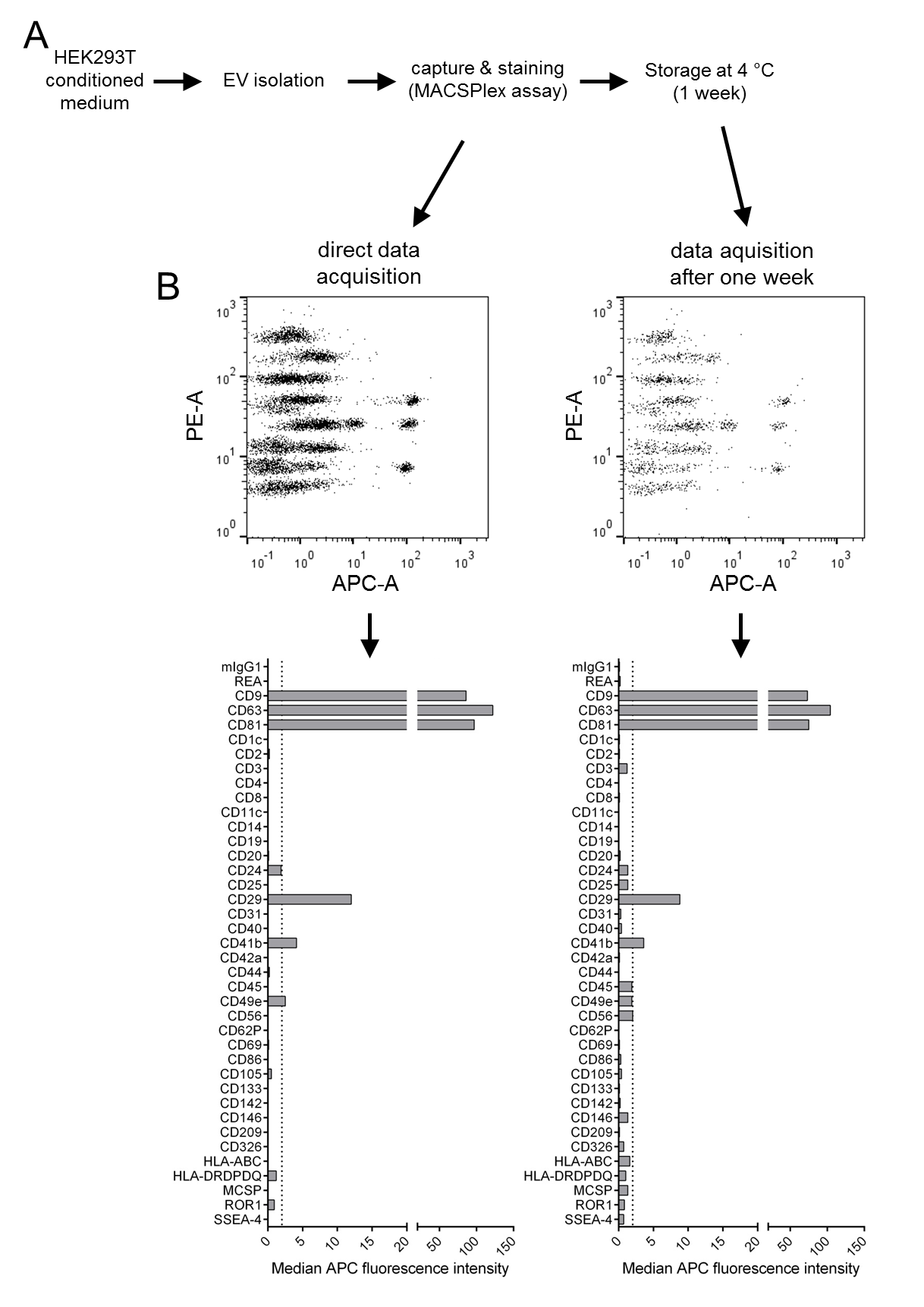


**Figure S4:**  **Samples are stable after preparation and before data acquisition.** Isolated HEK293T EVs were subjected to the default capture and staining protocol before either performing flow cytometric data acquisition directly or after storing the sample one week at 4 °C. This figure shows one representative example out of at least ten independently performed experiments.

**Figure S5 (related to Figure 4)**


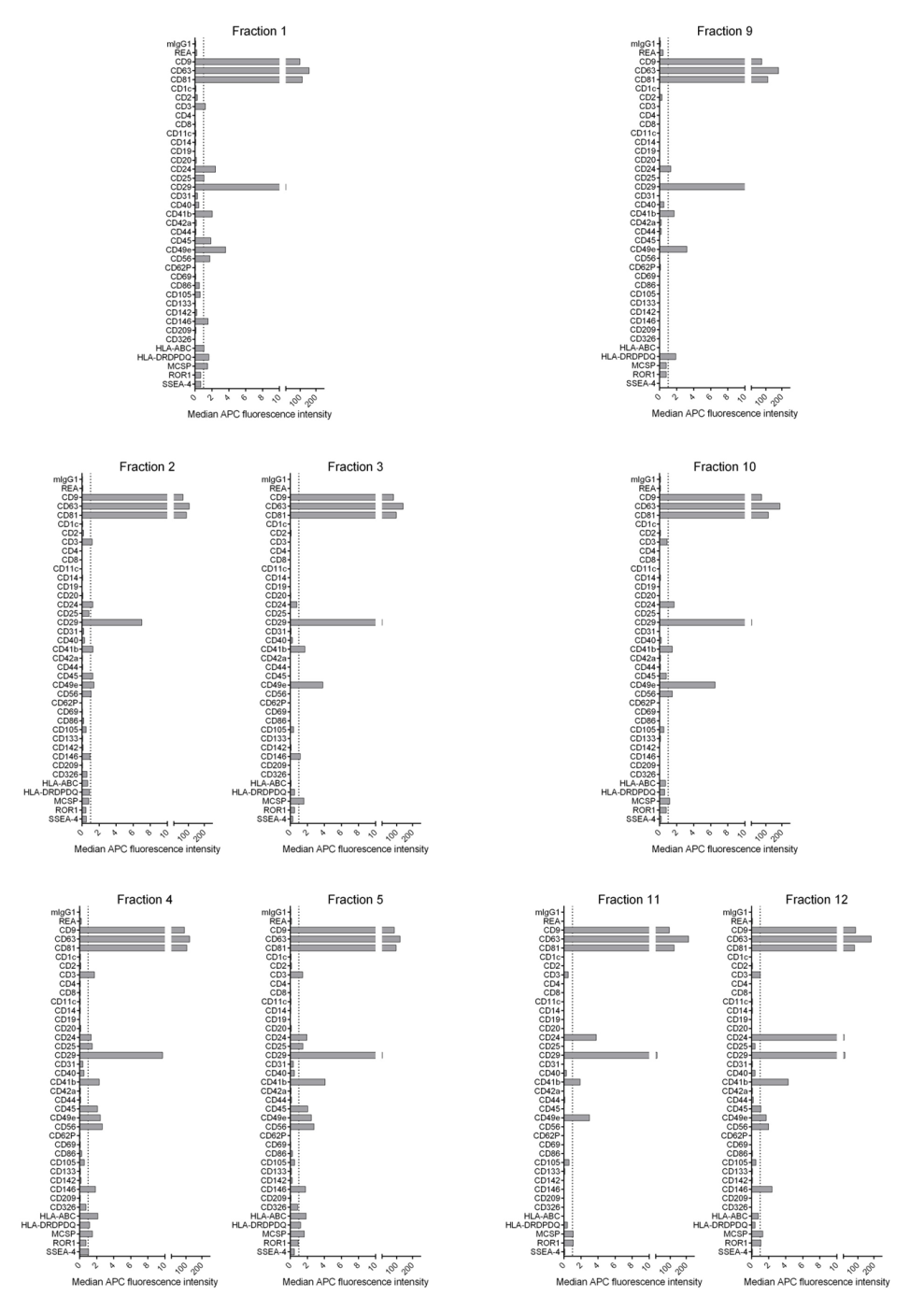


**Figure S5:**  **Assessment of EV surface signatures throughout different EV isolation protocols.** Complete datasets of fractions 1-5 and 9-12 as shown with reduced detail in Figure 4.

**Figure S6 (related to Figure 5)**

**
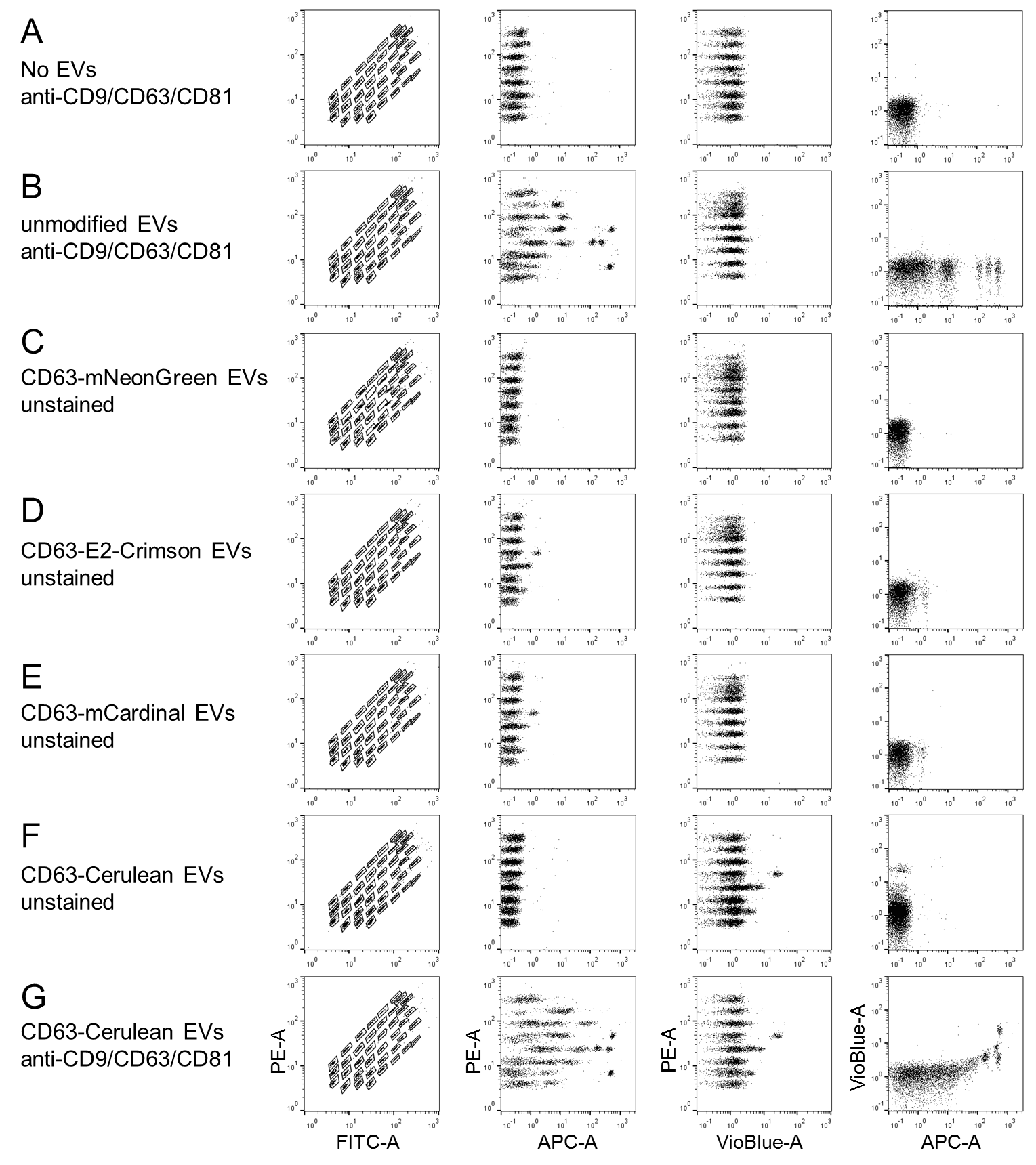
**

**Figure S6:**  **Assay compatibility with fluorescently-labeled EVs.** (A-G) Capture bead distribution for respectively stained or unstained samples as partly shown in Figure 5 with relevant channels, controls and combinations for all fluorescently labeled EVs are shown.

**Figure S7 (related to Figure 6)**

**
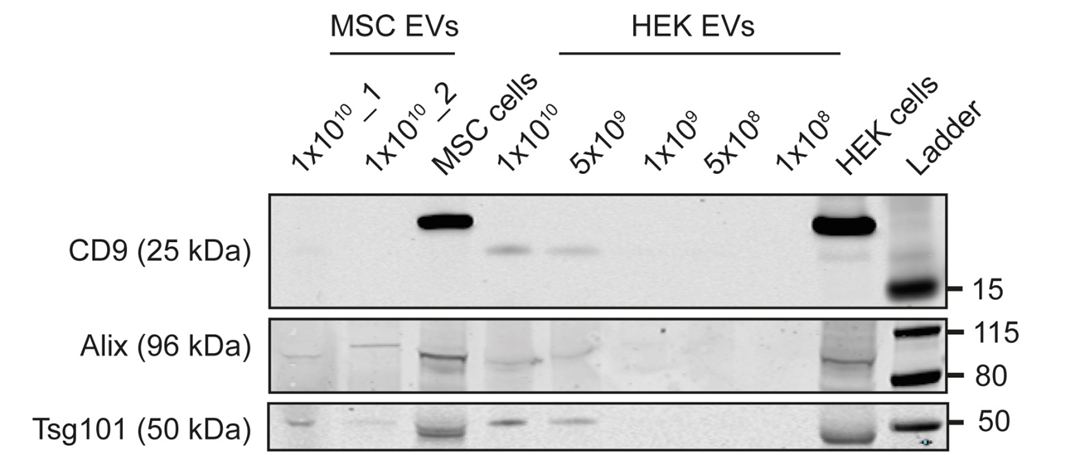
**

**Figure S7:**  **Detection of CD9, Alix and Tsg101 in cell lysates and EV preparations derived from HEK293T cells and MSCs by Western blot.** Two independent EV preparations were prepared from MSCs (1/2) and loaded at doses of 1x10^10^ EVs. HEK293T EVs were loaded at decreasing doses to estimate the detection limit.

**Figure S8 (related to Figure 8)**

**
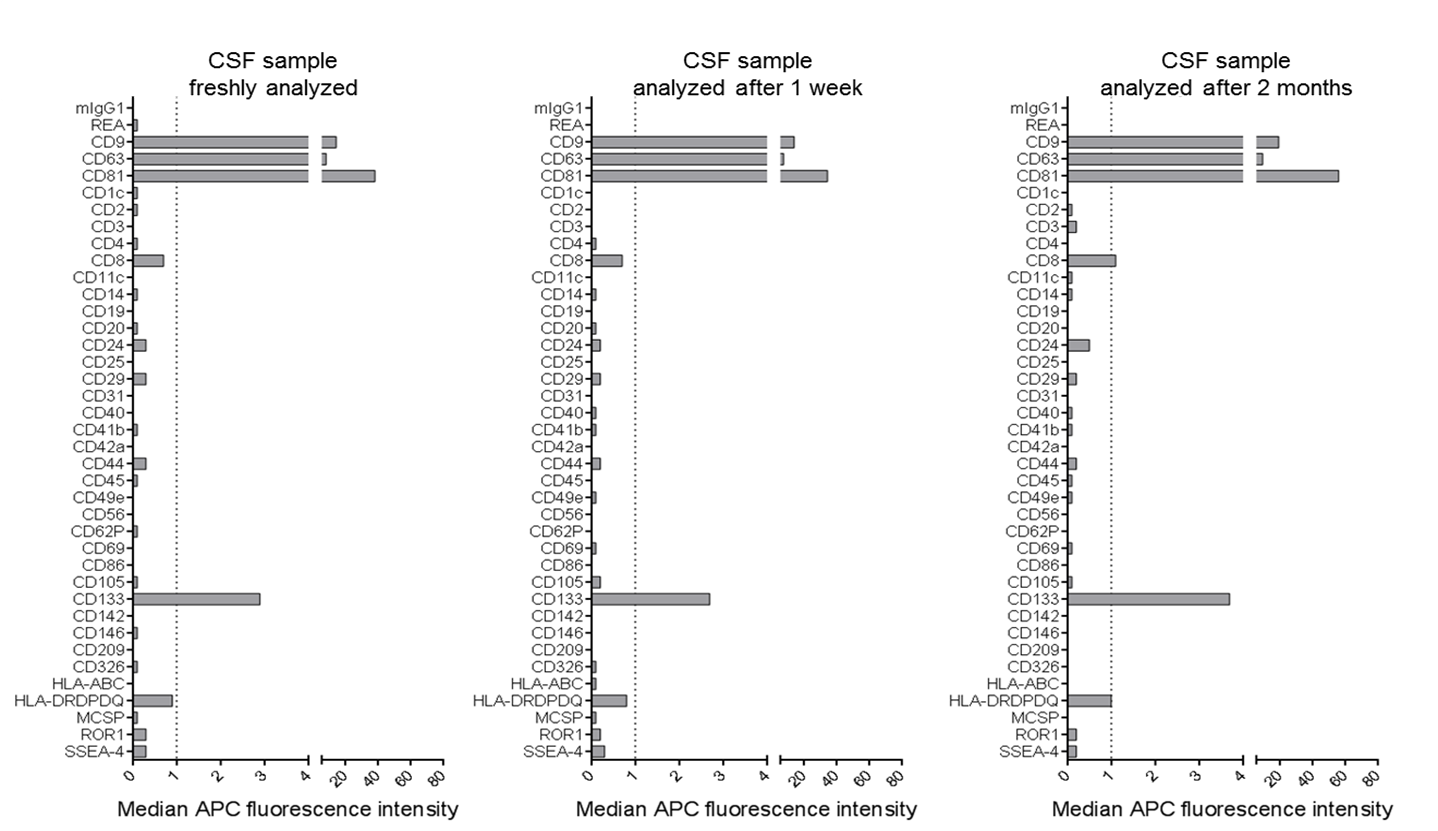
**

**Figure S8:**  **Sample stability of CSF samples.** A representative example of the same CSF sample is shown after direct capture and staining in the multiplex bead-based assay, or after 1 week stored at ‑20 ºC, or after one freeze-thaw cycle and total storage time of 2 months at ‑20 ºC. This general stability of signals was seen in at least 3 independent experiments when analyzing CSF samples derived from different donors.

**Figure S9 (related to Figure 8)**

**
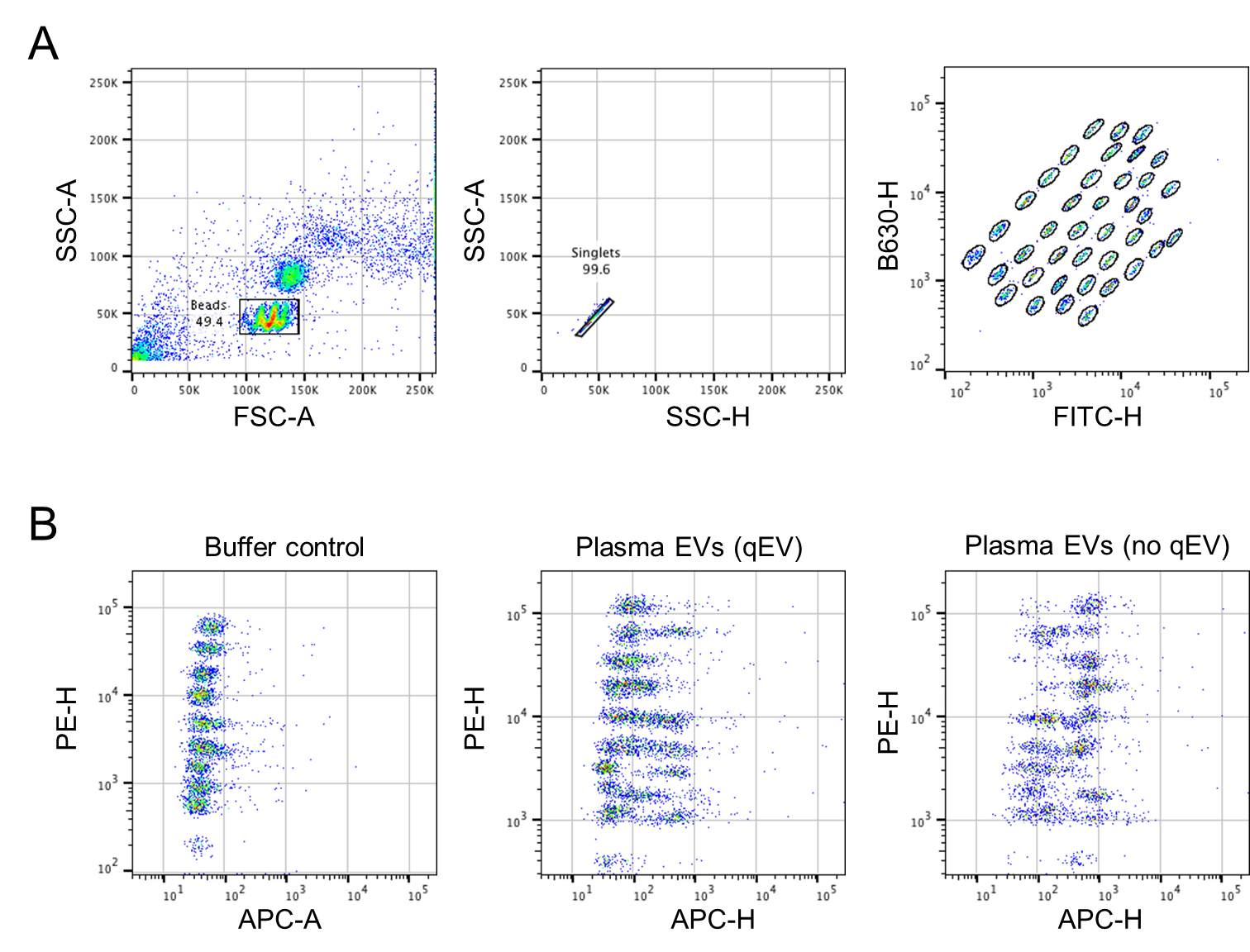
**

**Figure S9: Data analysis on the FACS Symphony A3 instrument.** (A) Gating strategy applied to identify single beads and capture bead subpopulations on the BD FACS Symphony A3 instrument. (B) Representative examples of a buffer control (samples treated the same but without EVs) and two plasma samples, purified via SEC (qEV) or not (no qEV), subjected to analysis via multiplex bead-based flow cytometry assay with an input dose of each 1x10^10^ EVs/assay.

**Figure S10 (related to Figure 8)**

**
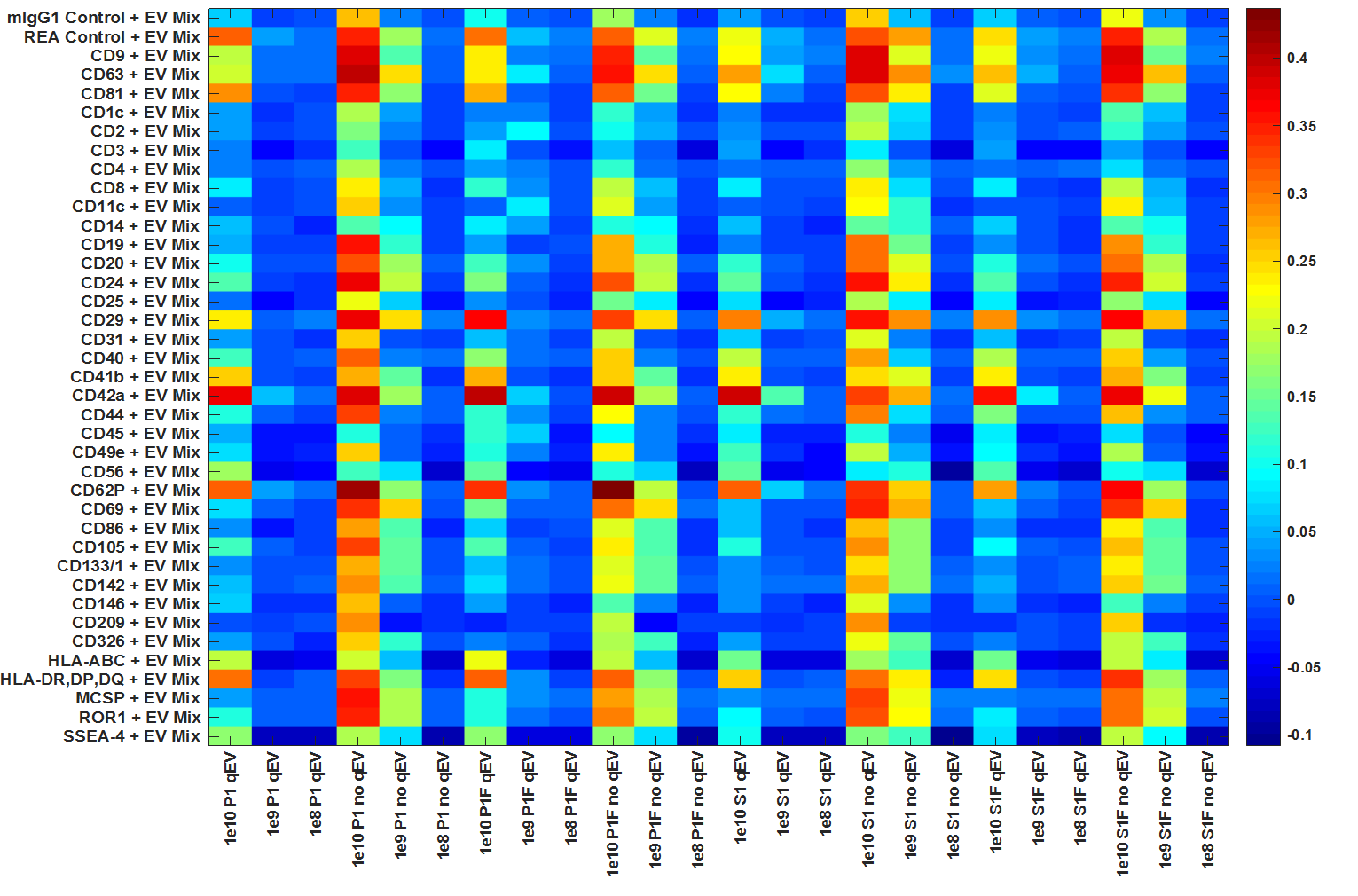
**

**Figure S10:**  **Comparison of sample parameters of blood-derived EVs.** Heatmap showing surface signatures obtained by bead-based multiplex flow cytometry for plasma (P1) and serum (S1), each with (qEV) or without (no qEV) EV isolation via qEV isolation columns. Samples were either measured freshly or after being frozen (F) before. Three different EV input amounts based on NTA were applied (10^8^, 10^9^, 10^10^). All samples were acquired on a FACS Symphony A3 instrument. Numbers are the Log10 fold increase of CD9+CD63+CD81-APC intensity for each capture bead population over control beads.

**Figure S11 (related to Figure 8)**

**
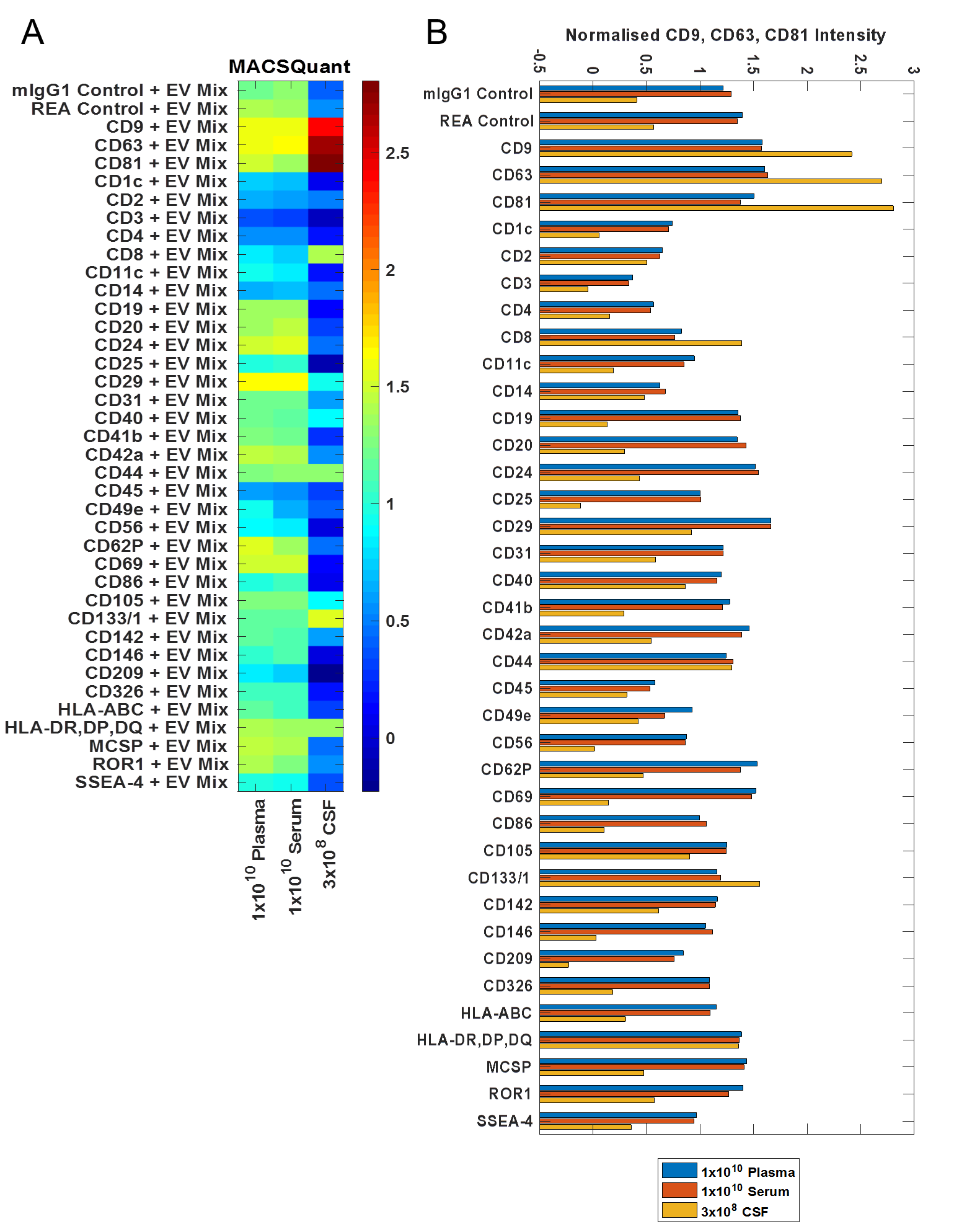
**

**Figure S11:**  **Normalisation of data acquired for EVS from different biological fluids on the MACSQuant.** (A) Heatmap and (B) corresponding bar graphs comparing EV surface signatures derived from analysis of EVs from plasma, serum (each with 1x10^10^ EVs input) and CSF (3x10^8^ EVs input).

**Figure S12 (related to Figure 8)**

**
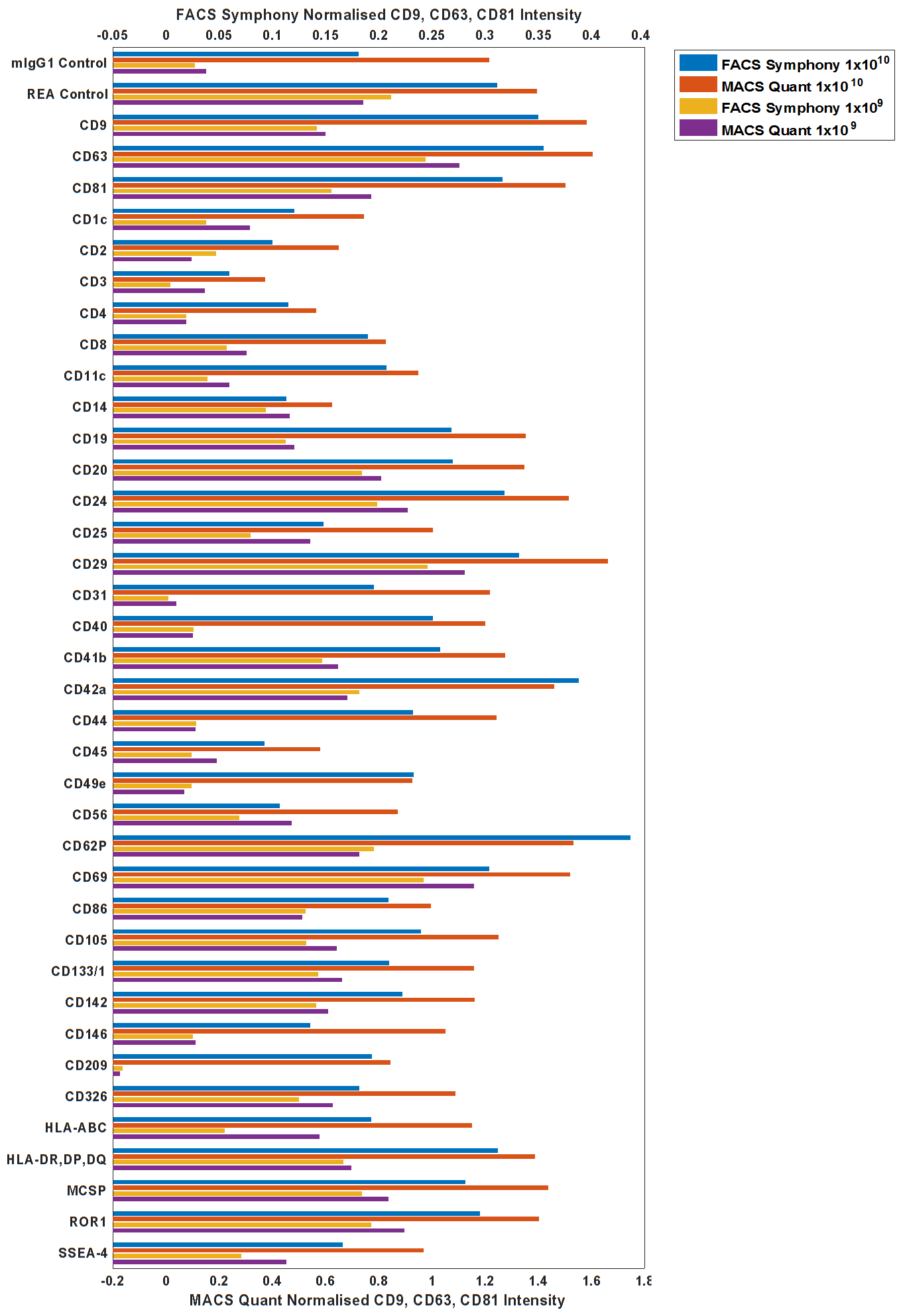
**

**Figure S12:**  **Normalisation of data acquired on different intruments.** The same plasma samples were acquired both at two different input doses (1x10^9^ or 1x10^10^ EVs) on a FACS Symphony and a MACSQuant instrument. All median intensity values were then normalized accordingly by background division to evaluate comparability of datasets acquired on different instruments.

**Figure S13 (related to Figure 9)**

**
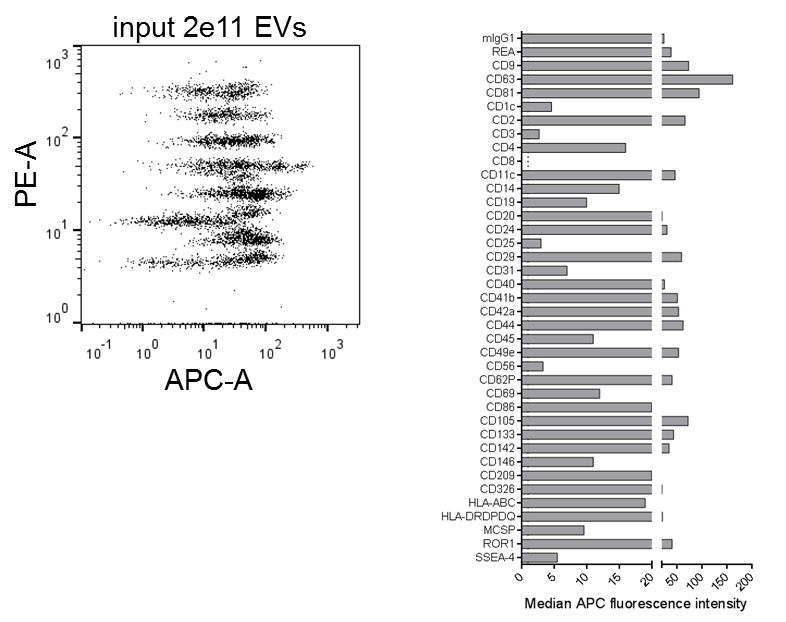
**

**Figure S13: Detection of injected human EVs in mouse plasma.** Dot plot and quantification of background-subtracted median APC fluorescence intensities from respective bead populations when using the dose of 2x10^11^ EVs (as injected per mouse) directly as input for the multiplex bead-based assay, which resulted in massive background signals also for internal controls.

**Figure S14 (related to Figure 10)**

**
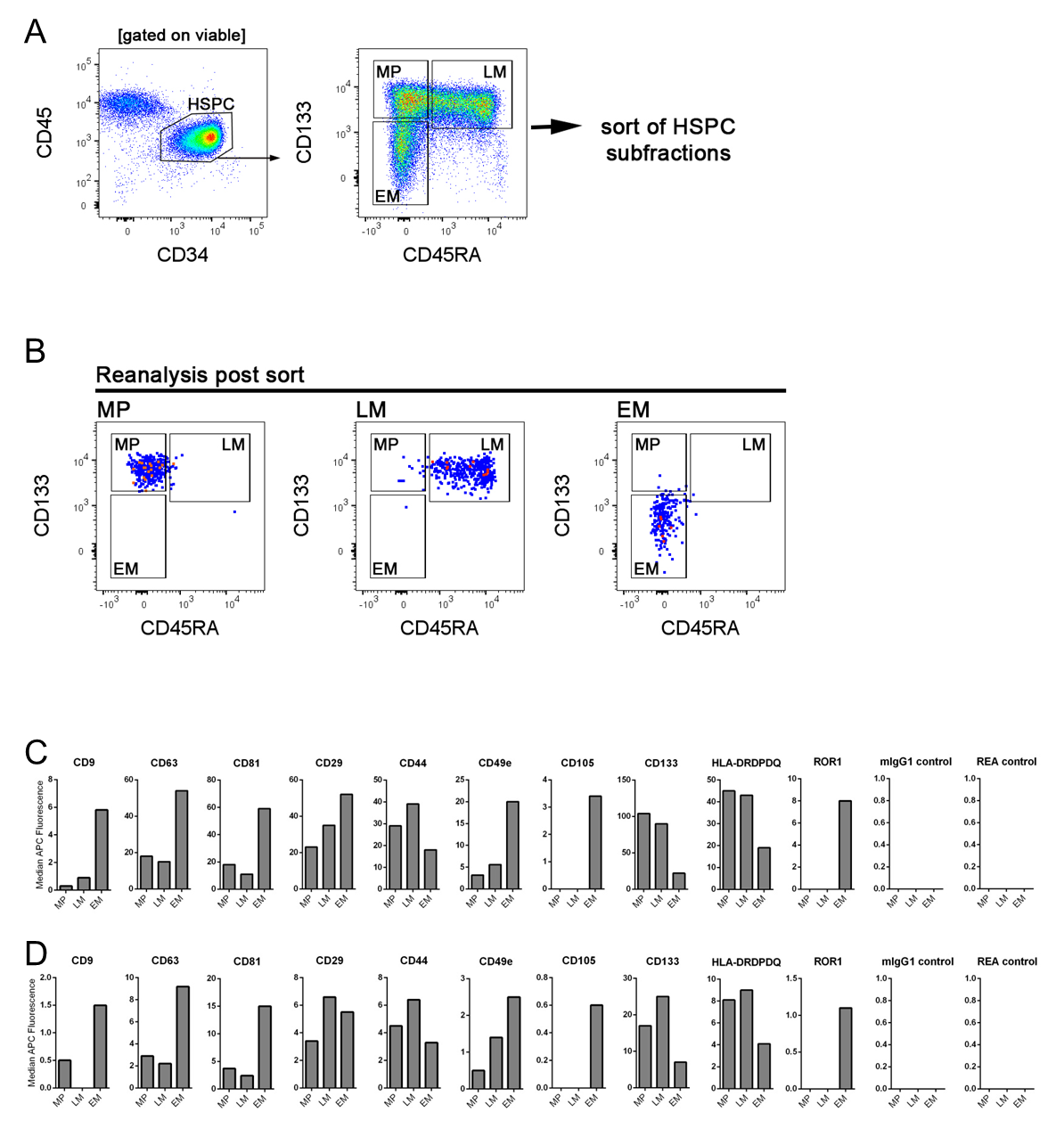
**

**Figure S14:**  **Analysis of EVs secreted by purified human hematopoietic stem/progenitor subsets.** (A) Gating strategy applied to identify and purify human hematopoietic stem and progenitor (HSPC) subfractions by flow cytometric cell sorting. After pregating on CD34(+)CD45(low) cells, multipotent progenitors (MP) were sorted as CD133(+)CD45RA(-), lympho-myeloid (LM) progenitors as CD133(+)CD45RA(+) and erythro-myeloid (EM) progenitors as CD133(low)CD45RA(-) as described before (Gorgens et al., 2013;Graffmann et al., 2015). (B) Reanalysis of sort-purified MP, LM and EM subfractions. (C/D) Expression of selected markers detected in conditioned medium derived from the 3 HSPC subfractions, shown for 2 independent experiments. The y-axis scaling was relatively adjusted for both experiments since overall signal intensities for the experiment shown in (D) was consistently 4-6 fold lower than the experiment shown in (C).

**Supplementary Tables**

**Table S1: MACSQuant Analyzer 10 instrument settings used in this study for multiplex bead-based EV analysis.** The trigger was set on FSC with a threshold of 116.

| **Laser** | **Parameter** | **Optical Filter (nm)** | **Designation** | **Voltage (V)** |
| --- | --- | --- | --- | --- |
| 488 nm | FSC | 488/10 |  | 488 |
| 488 nm | SSC | 488/10 |  | 575 |
| 488 nm | B1 | 525/50 | FITC | 440 |
| 488 nm | B2 | 585/40 | PE | 420 |
| 488 nm | B3 | 655-730 |  | 500 |
| 488 nm | B4 | 750LP |  | 500 |
| 635 nm | R1 | 655-730 | APC | 530 |
| 635 nm | R2 | 750LP |  | 530 |
| 405 nm | V1 | 450/50 | VioBlue | 500 |
| 405 nm | V2 | 525/50 |  | 500 |

**Table S2: Experimental details.** Supplemental details regarding sample preparation, assay protocols or acquisition details.

| **Figure** | **Details regarding sample preparation, assay protocols or acquisition details** |
| --- | --- |
| 1; S1; S2 | The CM of subconfluent HEK293T cells cultured for 48 hours was collected, pre-cleared by centrifugation (500 xg for 5 min, then 2,000 xg for 10 min) and filtered through 0.22 µm filters. The CM was then used without further dilution as assay input (120 µL CM + 15 µLl capture beads). For the medium control, 120 µL medium (OptiMem) was used accordingly. |
| 2 | To isolate EVs, the CM was first pre-cleared by centrifugation (500 xg for 5 min, then 2,000 xg for 20 min) and filtered through 0.22 µm filters. Then the CM was subjected to ultracentrifugation at 110,000 xg for 90 min with a second washing step applied as described in the material & methods section. The sample input of EVs per assay as indicated in Figure 2 was calculated based on particle counts assessed by NTA. |
| 3; S3;S4 | To isolate EVs, the CM was first pre-cleared by centrifugation (500 xg for 5 min, then 2,000 xg for 20 min) and filtered through 0.22 µm filters. Then the CM was concentrated and diafiltrated by TFF in 300 kDa hollow fiber filters and further concentrated by spin filtration with 10 kDa spin filters as described in the material & methods section. The normalized sample input of 5x 10^8^ EVs per assay was calculated based on particle counts assessed by NTA. |
| 4; S5 | All CM samples were pre-cleared by centrifugation (500 xg for 10 min, then 2,000 xg for 20 min). All following steps were done as indicated in Figure 4. The normalized sample input of 1x 10^8^ EVs per assay was calculated based on particle counts assessed by NTA. |
| 5; S6 | For all EV preparations used, the CM was first pre-cleared by centrifugation (500 xg for 5 min, then 2,000 xg for 20 min) and filtered through 0.22 µm filters. Then the CM was concentrated and diafiltrated by TFF in 300 kDa hollow fiber filters and further concentrated by spin filtration with 10 kDa spin filters as described in the material & methods section. The normalized sample input of 5x 10^8^ EVs per assay was calculated based on respective particle counts assessed by NTA. |
| 6; S7 | For HEK293T- and MSC-derived EV preparations, the CM was first pre-cleared by centrifugation (500 xg for 5 min, then 2,000 xg for 20 min) and filtered through 0.22 µm filters. Then the CM was concentrated and diafiltrated by TFF in 300 kDa hollow fiber filters and further concentrated by spin filtration with 10 kDa spin filters as described in the material & methods section. All sample inputs were calculated based on respective particle counts assessed by NTA. |
| 7 | The CM of subconfluent PANC-1 or IGROV1 cells cultured for 48 hours in OptiMem was collected, pre-cleared by centrifugation (500 xg for 10 min, then 2,000 xg for 20 min) and filtered through 0.22 µm filters. The CM was then used without further dilution as assay input (120 µL CM + 15 µL capture beads). Medium controls (120 µL medium + 15 µL capture beads) were used for background subtraction of bead population MFI values. 5 µL FolR1 antibodies were used per assay to detect FolR1 positive EVs. |
| 8; S8-S12 | Plasma/serum samples were analyzed with the following modifications applied to the bead-based multiplex assay procedure: Either 10^8^, 10^9^, or 10^10^ EVs were added to 0.22 µm filter plates from either plasma or serum that had been purified with SEC (qEV) or not. Samples that were not run through SEC columns were assumed to be ten times more concentrated than those that were. MBP buffer was added to a final volume of 150 µL before 10 µL of capture beads were added. Samples were incubated, washed and stained according to the default plate protocol described in the material & methods section. Samples were then transferred to FACS tubes for analysis by flow cytometry and analysed with a FACS Symphony A3 (see Table S3 for details). |
| 9; S13 | To isolate MSC-EVs, the CM was first pre-cleared by centrifugation (500 xg for 5 min, then 2,000 xg for 20 min) and filtered through 0.22 µm filters. Then the CM was concentrated and diafiltrated by TFF in 300 kDa hollow fiber filters and further concentrated by spin filtration with 10 kDa spin filters as described in the material & methods section. The injection volumes and the respective EV input doses per assay was calculated based on respective particle counts assessed by NTA. |
| 10; S14 | CM samples were pre-cleared by centrifugation at 900 xg for 5 min and 2,000 xg for 5 min. No filtration was applied due to limiting sample volumes. Samples were frozen at -80 °C for 1-3 weeks before they were thawed and analyzed in the multiplex bead-based assay with 80 µL CM input per assay (+ 15 µL capture beads). All further experimental details are given in the main figure legend and the material & methods section. |

**Table S3: FACS Symphony A3 instrument settings used in this study for multiplex bead-based EV analysis.**

| **Laser Wavelength** | **Laser  Power** | **Parameter** | **Optical Filter (nm)** | **Detector Voltage (V)** |
| --- | --- | --- | --- | --- |
| 488 nm | 200 mW | FSC | 488/10 | 450 |
| 488 nm | 200 mW | SSC | 488/10 | 150 |
| 488 nm | 200 mW | FITC-H | 515/20 | 300 |
| 488 nm | 200 mW | BB630-H | 610/20 | 420 |
| 640 nm | 200 mW | APC-H | 670/30 | 400 |

**Supplementary References**

Gorgens, A., Radtke, S., Mollmann, M., Cross, M., Durig, J., Horn, P.A., and Giebel, B. (2013). Revision of the human hematopoietic tree: granulocyte subtypes derive from distinct hematopoietic lineages. *Cell Rep* 3**,** 1539-1552.

Graffmann, N., Brands, J., Gorgens, A., Vitoriano Da Conceicao Castro, S., Santourlidis, S., Reckert, A., Michele, I., Ritz-Timme, S., Fischer, J.C., Adjaye, J., Kogler, G., Giebel, B., and Uhrberg, M. (2015). Age-Related Increase of EED Expression in Early Hematopoietic Progenitor Cells is Associated with Global Increase of the Histone Modification H3K27me3. *Stem Cells Dev* 24**,** 2018-2031.
